# *ChemEnv*: a fast and robust coordination environment identification tool

**DOI:** 10.1107/S2052520620007994

**Published:** 2020-07-21

**Authors:** David Waroquiers, Janine George, Matthew Horton, Stephan Schenk, Kristin A. Persson, Gian-Marco Rignanese, Xavier Gonze, Geoffroy Hautier

**Affiliations:** a Institute of Condensed Matter and Nanosciences, Université catholique de Louvain, Chemin des Étoiles 8, 1348 Louvain-la-Neuve, Belgium; b Energy Technologies Area, Lawrence Berkeley National Laboratory, Berkeley, CA 94720, USA; c Department of Materials Science and Engineering, University of California, Berkeley, CA 94720, USA; d BASF SE, Digitalization of R&D, Carl-Bosch-Str. 38, 67056 Ludwigshafen, Germany; e Skolkovo Institute of Science and Technology, Skolkovo Innovation Center, Nobel St. 3, Moscow, 143026, Russia

**Keywords:** coordination environment, continuous symmetry measure, Voronoï, coordination number

## Abstract

Coordination or local environments have been used to describe, analyze and understand crystal structures for more than a century. Here, a new tool called *ChemEnv*, which can identify coordination environments in a fast and robust manner, is presented. In contrast to previous tools, the assessment of the coordination environments is not biased by small distortions of the crystal structure. Its robust and fast implementation enables the analysis of large databases of structures. The code is available open source within the *pymatgen* package and the software can also be used through a web app available on http://crystaltoolkit.org through the Materials Project.

## Abstract 2

A new tool called *ChemEnv*, which can identify coordination environments in a fast and robust manner, is presented.

## 1. Introduction   

Inorganic crystal structures are typically described by their structure prototype or by a more local concept of ‘coordination environment’ (Müller, 2007 ▸; Allmann & Hinek, 2007 ▸). Coordination environments or local environments (*e.g.* octahedral, tetrahedral, *etc.*) are often used in structure visualization as they clarify the crystal arrangement. These environments can also be used to understand crystal structures and their properties. P. Pfeiffer was the first to transfer this concept of coordination environments from coordination complexes to crystals to rationalize crystals as large molecules (Pfeiffer, 1915 ▸, 1916 ▸). Very often these coordination environments are determined in a non-automatic manner by the individual researcher. Local environments play a major role in solid state chemistry and physics as well as materials science. For instance, the famous Pauling rules, which have been used to understand and rationalize crystal structures for 90 years, rely heavily on this concept (Pauling, 1929 ▸). In the Pauling rules, the analysis of the coordination environments is used to determine the stability of a material. Electronic, optical, magnetic and other properties of crystals have also been related to and explained by local environments (Hoffmann, 1987 ▸, 1988 ▸; Lueken, 2013 ▸; Peng *et al.*, 2015 ▸). In recent years, coordination environments have been discussed and used as structural descriptors to derive structure–property relationships via machine-learning methods (Jain *et al.*, 2016 ▸; Zimmermann *et al.*, 2017 ▸). Some of us have analyzed the coordination environments present in oxides in a statistical manner (Waroquiers *et al.*, 2017 ▸). Such large-scale analyzes require an easily reproducible, robust and automatic determination of coordination environments. Since the transfer of the concept of coordination environments from coordination complexes to crystals, various approaches to determine coordination numbers, coordination environments, or the distortion of coordination environments have been developed (Frank & Kasper, 1958 ▸; Brunner & Schwarzenbach, 1971 ▸; Carter, 1978 ▸; O’Keeffe, 1979 ▸; Hoppe, 1979 ▸; Pinsky & Avnir, 1998 ▸; Guńka & Zachara, 2019 ▸; Stoiber & Niewa, 2019 ▸). However, the methods mentioned so far are not well suited for a robust and automatic assessment of coordination environments in very large databases consisting of several thousands of crystal structures such as the Inorganic Crystal Structure Database (Bergerhoff & Brown, 1987 ▸, Zagorac *et al.*, 2019 ▸), Pearson’s database (Villars & Cenzual, 2018 ▸) or the Cambridge Structural Database (Groom *et al.*, 2016 ▸). Indeed, some of these methods can be sensitive to small distortions due to predefined cut-offs while others rely on additional chemical information that is not directly available from the sole consideration of the geometry of the crystal. Moreover, some of these methods only deal with the identification of the coordination number without assigning a specific environment to a given site. To fill this gap we developed *ChemEnv*, a fast and robust tool to identify coordination environments. It has already been applied in the study of coordination environments of oxides (Waroquiers *et al.*, 2017 ▸) and in a rigorous assessment study of the Pauling rules (George *et al.*, 2020 ▸). It is embedded in *pymatgen* – a Python library for materials analysis which is part of the Materials Project that aims at the accelerated design of new materials (Ong *et al.*, 2013 ▸; Jain *et al.*, 2013 ▸). Our approach relies on the similarity of such distorted polyhedra present in the crystal structure to ideal reference polyhedra. After a neighbor analysis, we identify potential local environments and compare them through a distance metric to a database of perfect local environments. Different algorithms called strategies are then used to decide on a local environment assignment and the final result can present a unique environment or a mixture of several environments. This approach which is robust to distortion will be described in detail in this paper.

## 2. Method/algorithm   

### 2.1. Aspects of coordination environments identification   

In the process of identifying coordination environments of a given atom, two main questions have to be considered:

(*a*) What are the neighbors of this atom?

(*b*) What is the overall arrangement of these neighbors around this atom?

The first question refers to what is called the coordination number while the second corresponds to the coordination or local environment. The answer to these questions is very clear when the local structure of the atom is close to a perfect environment. However, when relatively large distortions are present, the identification can be much more difficult. In particular, a given local environment can be identified as a *mix* of two or more coordination environments (which can be of the same coordination number or not).

### 2.2. Voronoï analysis   

The neighbors of a given atom in a given structure are determined using a modified Voronoï approach similar to what was proposed by O’Keeffe (1979 ▸). The Voronoï analysis allows for the splitting of the space into regions that are closer to one atom than to any other one. In the standard Voronoï approach for determining the neighbors of a given atom *X*, all the atoms {*Y*_1_,…,*Y*_*n*_} whose regions are contiguous to the region of atom *X* are considered as coordinated to atom *X*. The distances between atom *X* and each of its neighbors are written 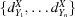. The common faces 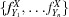 between the region of atom *X* and each of the regions of atoms {*Y*_1_,…,*Y*_*n*_} define solid angles 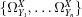 subtended by these faces at atom *X*.

**Figure 1:**
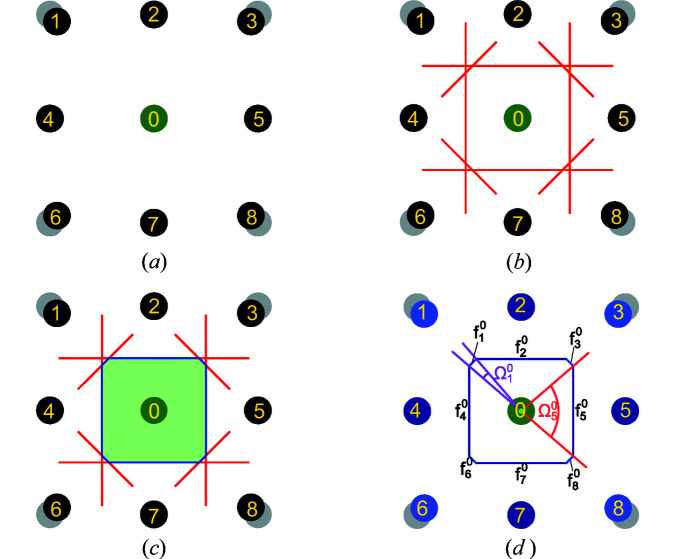
Voronoï construction.

The Voronoï regions are easily understood by drawing the perpendicular area bisectors for each pair of atoms *X* and *Y*. Fig. 1 ▸ illustrates the concept in two dimensions (in which area bisectors are thus replaced by line bisectors). The example shown is a slightly distorted square lattice [see Fig. 1 ▸(*a*)] where the atoms at the corners (atoms 1, 3, 6 and 8) are displaced towards the central green atom (atom 0). The perfect square lattice is shown by the gray atoms. In Fig. 1 ▸(*b*), the perpendicular line bisectors (in red) are drawn for each segment from the central (green) atom and all other (black) atoms around it. The Voronoï region of the central atom corresponds to the region in light green in Fig. 1 ▸(*c*). Fig. 1 ▸(*d*) shows the faces 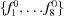 attributed to each pair of atoms *0*–*i* with *i* = 1…8. The solid angle is illustrated for neighbors 1 and 5 by 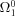 and 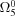, respectively.

**Figure 2:**
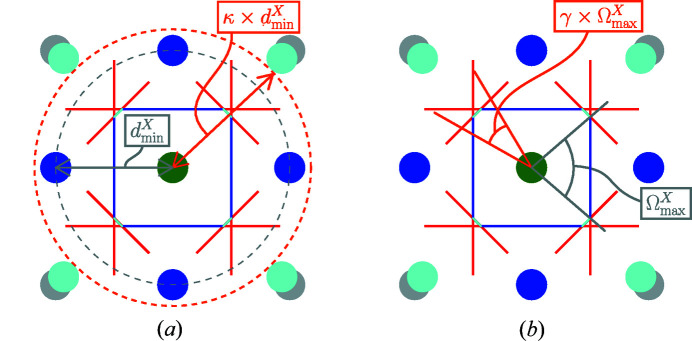
Schematic representation of the cut-off parameters used in the Voronoï analysis of neighbors: (*a*) distance cut-off and (*b*) angle cut-off. (*a*) Distance cut-off parameter κ. 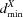 is the distance to the closest neighbor (one of the dark blue atoms). Any atom that lies outside the sphere of radius 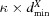 (in dashed orange) is not considered as a coordinated neighbor. Atoms at the corner (in light blue) are not considered as neighbors. (*b*) Angle cut-off parameter γ. 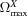 is the largest solid angle to a neighbor atom. Any atom for which the solid angle is smaller than 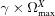 (in orange) is not considered as a coordinated neighbor. Atoms at the corner (in light blue) are not considered as neighbors. [Adapted with permission from D. Waroquiers *et al.* (2017 ▸).]

In our modified approach, two additional cut-offs can be added as shown schematically in two dimensions in Fig. 2 ▸:

(*a*) The first cut-off excludes neighbors on the basis of the distance [Fig. 2 ▸(*a*)]. Let 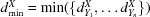 be the distance to the closest neighbor of atom *X* and κ ≥ 1.0 be the distance cut-off parameter. All atoms lying inside the sphere of radius 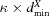 are considered as coordinated neighbors while those lying outside are disregarded. We define the *normalized distance* 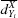 of each neighbor *Y*_*i*_ as 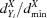.

(*b*) The second cut-off is based on the solid angles 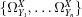 introduced before [Fig. 2 ▸(*b*)]. Let 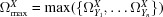 be the biggest solid angle to a neighbor for atom *X* and γ ≤ 1.0 be the angle cut-off parameter. All neighboring atoms with a solid angle smaller than 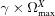 are not considered as coordinated to atom *X*. We define the *normalized angle* 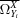 of each neighbor *Y*_*i*_ as 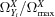.

It is possible to use both cut-offs at the same time in which case a given atom is not considered as a coordinated neighbor if either one of the cut-offs disregards it as a coordinated neighbor.

The modified Voronoï procedure presented above allows for the determination of the coordinated neighbors of a given atom *X* for a given set of distance/angle parameters. The identification of the coordinated neighbors of atom *X* defines the *local environment* of this atom. The identification of the model environment which this local environment resembles the most is described in the next section.

### 2.3. The *shape* recognition problem and the continuous symmetry measure   

**Figure 3:**
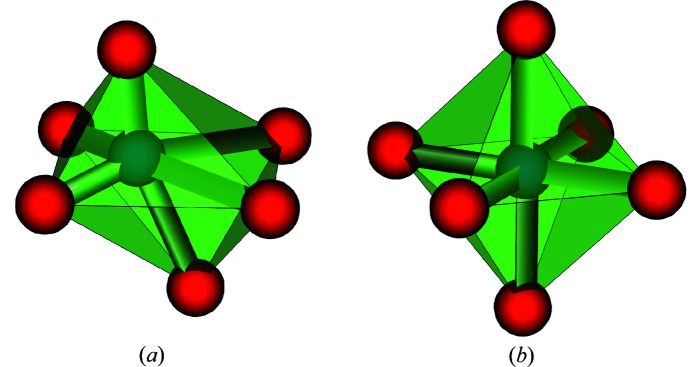
The *shape* recognition problem. It consists in identifying whether the distorted octahedron in (*a*) is more similar to the perfect (model) octahedron in (*b*) than to any other model polyhedron. This presupposes that there exists a list of model polyhedra to be compared to.

The *shape* recognition problem consists in the identification of the model environment to which a local and possibly distorted environment resembles the most. Fig. 3 ▸ illustrates this problem. A distorted octahedron is shown in Fig. 3 ▸(*a*). Whether this distorted octahedron is more similar to a perfect octahedron [see Fig. 3 ▸(*b*)] than to any other (model) shape is precisely the purpose of the shape recognition. This inherently implies that a list of model polyhedra to be compared to is known *a priori*. We stick to the list of coordination environments recommended by the IUPAC (Hartshorn *et al.*, 2007 ▸) and by the IUCr (Lima-de Faria *et al.*, 1990 ▸). This list of environments, their symbol, coordinates and additional *meta*-information are given as supplementary information.

In order to *measure* the closeness of a local environment to each perfect model environment, the *Continuous Symmetry Measure* (CSM) is used, as proposed by Pinsky & Avnir (1998 ▸). This CSM can be interpreted as a measure of similarity between shapes. For a given structure 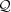 composed of 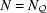 atoms (vertices) with coordinates {**q**_*k*_, *k* = 1, 2, …, *N*}, the CSM 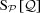 with respect to a model polyhedron 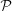 with 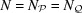 vertices {**p**_*k*_, *k* = 1, 2, …, *N*} is defined as: 
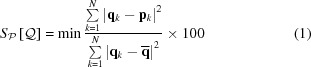
with 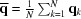.

With this definition, the value of the CSM is guaranteed to be in the [0.0, 100.0] interval. A value of 0.0 for the CSM indicates that the two shapes are identical, *i.e.* the structure 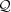 corresponds to the perfect structure 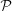. Instead, when the structure is distorted, the value of the CSM gives a degree of distortion of the structure 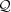 with respect to the perfect structure 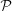. As such, the CSM can be understood as one definition of a *distance to a shape*.

**Figure 4:**
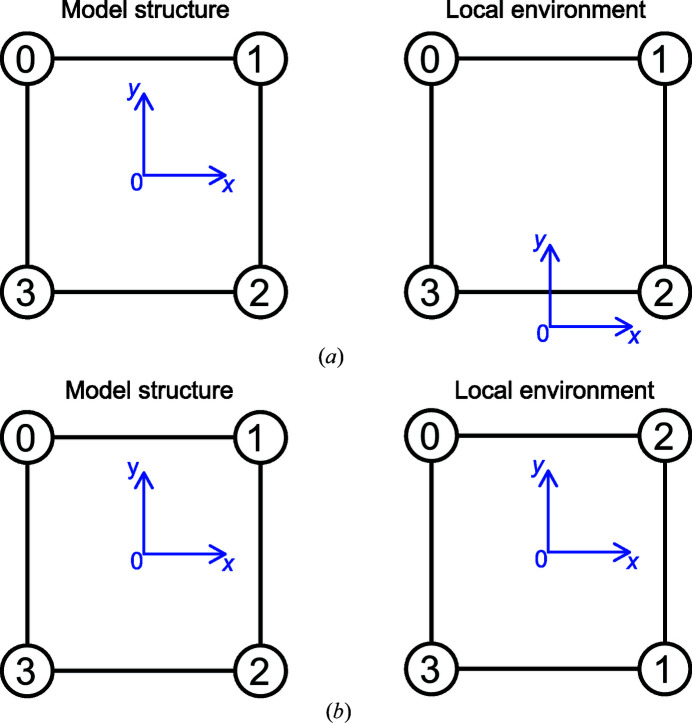
Translational and ordering degrees of freedom for the minimization in equation (1). (*a*) Translation of the polyhedron and (*b*) ordering of the vertices.

**Figure 5:**
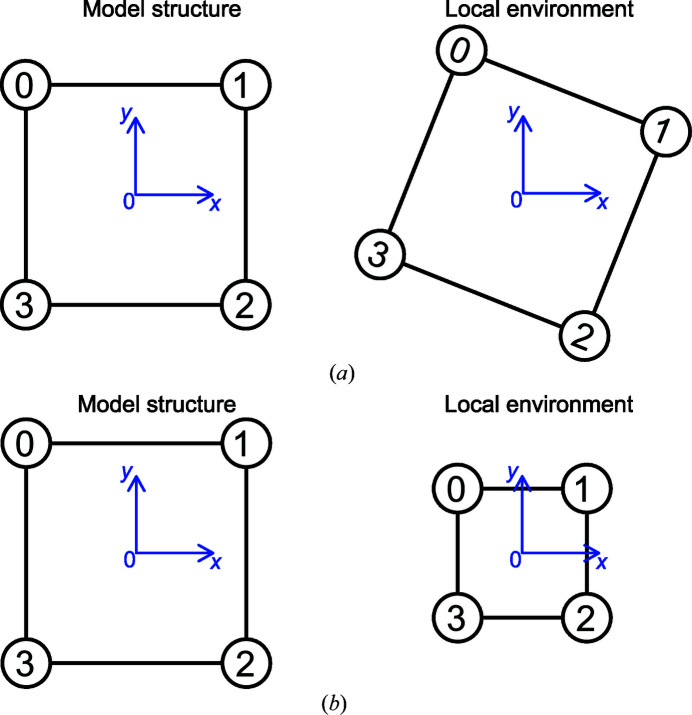
Rotational and size degrees of freedom for the minimization in equation (1). (*a*) Orientation and (*b*) size.

In equation (1), the minimization has to be performed with respect to four different degrees of freedom:

(i) *Translation* [see Fig. 4 ▸(*a*)]. This minimization is easily addressed by translating the local structures to their center of mass.

(ii) *Ordering* of the atoms [see Fig. 4 ▸(*b*)]. The simplest method is to test all possible permutations of indices. This guarantees a correct value for the CSM but the number of permutations scales as *N*! making it time-consuming for large (*N* > 6) coordination numbers. The symmetry of the model polyhedra is used to reduce the number of independent permutations for *N* ≤ 6. For larger *N*, a different approach is adopted (see Section 2.4).

(iii) *Orientation* of the structure [see Fig. 5 ▸(*a*)]. The local (distorted) structure is rotated in order to minimize the numerator in equation (1) by using an alignment procedure based on the singular value decomposition (Kabsch, 1976 ▸; Kabsch, 1978 ▸).

(iv) *Size* of the structure [see Fig. 5 ▸(*b*)]. A scaling factor is applied to the local structure to avoid size effects: the local structure is normalized to the root-mean square distance from the center of mass of the structure to all vertices.

**Figure 6:**
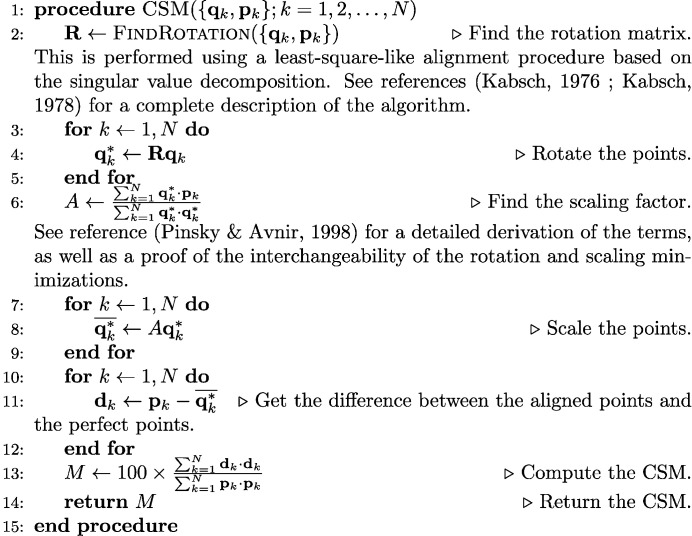
Algorithm 1. Computation of the CSM for a given permutation.

The minimization process presented above is equivalent to the point set registration algorithms used in shape or pattern recognition (Pomerleau *et al*., 2015 ▸). The main challenge comes from the fact that the correspondence between points in 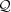 and 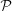 (*i.e.* the ordering problem described above) is unknown. In pattern recognition in which the number of points is usually large, algorithms based on pair correlation functions combined with statistical analysis are widely used [see Maiseli *et al.* (2017 ▸) and references therein]. In contrast, for small number of points, a different approach has to be adopted. As briefly outlined above, the simplest solution (which is used for *N* ≤ 6) is to test all possible permutations of indices (ignoring symmetrically identical ones), while for larger *N* the number of permutations is reduced using the *separation-plane algorithm* (see Section 2.4). In any case, for a given permutation of points, the CSM can be obtained thanks to algorithm 1 (see Fig. 6 ▸, points in 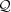 have been translated such that their center of mass coincide with that of 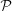). The exact CSM is then the smallest one of all the CSM computed for each permutation considered.

### 2.4. Separation-plane algorithm   

When the number *N* of coordinated neighbors increases, the number of permutations needed to minimize equation (1) scales as *N*!. When the correspondence of vertices between the local distorted structure and the perfect model polyhedron is not known (which is usually the case for the application of the procedure to large databases of structures), this makes the computation of the CSM almost infeasible for *N* > 10 and very time-consuming for 6 > *N* ≥ 10 with the standard procedure (*e.g.* 9! = 362 880, 12! = 479 001 600).

**Figure 7:**
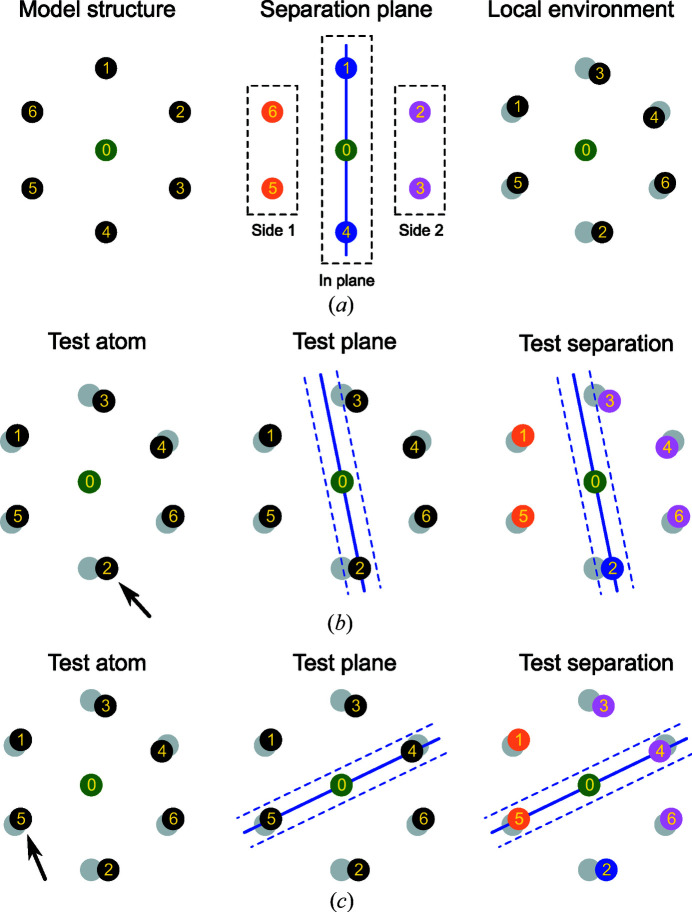
Illustration of the separation plane algorithm. (*a*) Model and local (distorted) structure, (*b*) first trial for separation plane algorithm and (*c*) second trial for separation plane algorithm.

In order to overcome this difficulty, the separation plane algorithm has been devised to drastically reduce the computational time needed. The basic idea is to identify possible planes in the distorted structure that can be assigned to a plane in the model polyhedron in order to reduce the number of permutations needed to find the right correspondence between points and hence the correct CSM. This idea is illustrated in Fig. 7 ▸ for a two-dimensional case. The points of the perfect model shape are separated into three different groups: the set of points supposed to lie within the plane and the two sets of points on either side of the plane. The permutation space is thus reduced because *N*! is always larger than *N*_1_!*N*_2_!*N*_3_! if at least two of *N*_1_, *N*_2_, *N*_3_ are larger than or equal to 1. For the example in Fig. 7 ▸, the number of permutations is reduced from 6! = 720 to 2! × 2! × 2! = 8. Additionally, for larger environments in which the separating plane contains more than three points, these can be ordered using clockwise or counterclockwise ordering, hence reducing the number of permutations even further.

**Figure 8:**
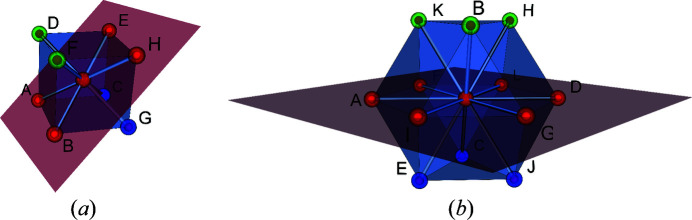
Examples of separation planes. (*a*) Separation (2, 4, 2) in the cubic environment: points A, B, H and E (in red) belong to the plane that separates points D and F (in green) from points C and G (in blue). (*b*) Separation (3, 6, 3) in a cuboctahedron: points A, I, G, D, L and F (in red) belong to the plane that separates points C, E and J (in blue) from points H, K and B (in green).

A *separation* is defined by its separation plane *P*_*perf*_ passing through at least three points of the perfect polyhedron 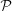 and by the two separated groups of points *S*_*perf*_ and *T*_*perf*_ located on either side of the plane. The set of points in the plane is written as *P* = {**p**_*j*_, *j* = 1,…*N*_*P*_} while *S* = {**s**_*m*_, *m* = 1, …*N*_*S*_} and *T* = {**t**_*n*_, *n* = 1, …*N*_*T*_} stand for the two sets of points on either side of the plane. By construction, {**q**_*k*_} = {**p**_*j*_} ∪ {**s**_*m*_} ∪ {**t**_*n*_} and *N* = *N*_*P*_ + *N*_*S*_ + *N*_*T*_. We use ϒ_*perf*_ = (*N*_*S*_, *N*_*P*_, *N*_*T*_) as an abridged notation for the separation. For the example illustrated in Fig. 7 ▸, the separation is noted (2, 2, 2). An illustration of two separation planes for the cubic and cuboctahedral environments is provided in Fig. 8 ▸.

**Figure 9:**
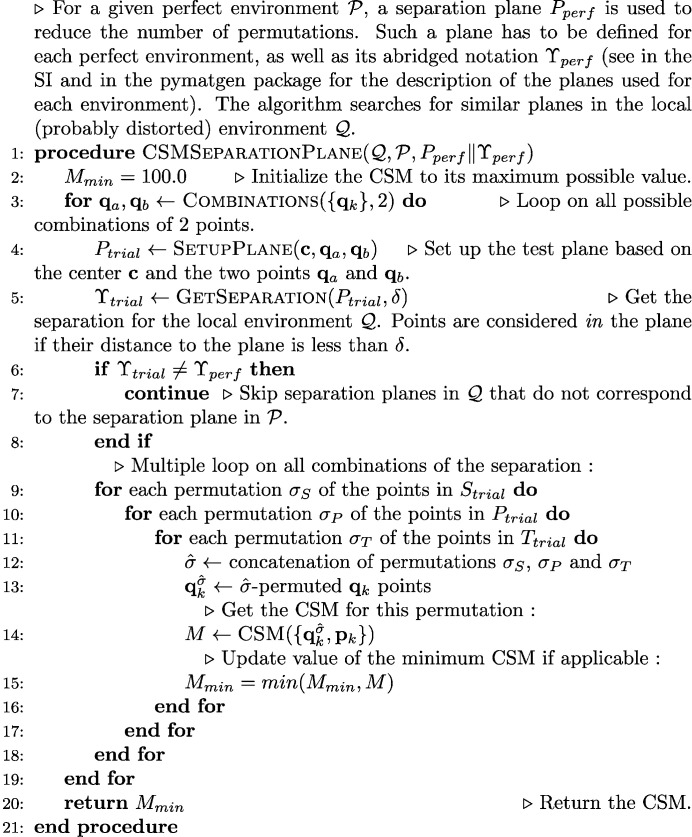
Algorithm 2. The separation plane algorithm.

The procedure for the computation of the CSM of environments with more than six atoms is described in algorithm 2 which is shown in Fig. 9 ▸. Separation planes have been defined for all the perfect model environments above six atoms. Usually, more than one separation plane can be defined in a given model polyhedron. In practice, the overall algorithm tests all the available separation planes that have been defined for the polyhedron under consideration. The list of separation planes for each coordination environment is available as SI and is also easily viewable with a script provided in the *ChemEnv* subpackage of *pymatgen*.

The algorithm has been optimized by ordering the points of the separation plane in a clockwise or counterclockwise direction whenever possible. This makes it possible to reduce the number of permutations related to the separation plane. For example, for the separation (3, 6, 3) of the cuboctahedron shown in Fig. 8 ▸(*b*), the number of permutations of the points in the plane is 6! = 720. Ordering the points in the perfect and local environments makes it possible to reduce the number of trials to six. A similar optimization is also possible for the two separated groups of points for the separations in which these groups contain a sufficient number of points (*e.g.* in the icosahedral environment, the separation plane contains four points and splits the other points into two groups of four points each).

### 2.5. Neighbor sets and distance/angle parameters maps   

**Figure 10:**
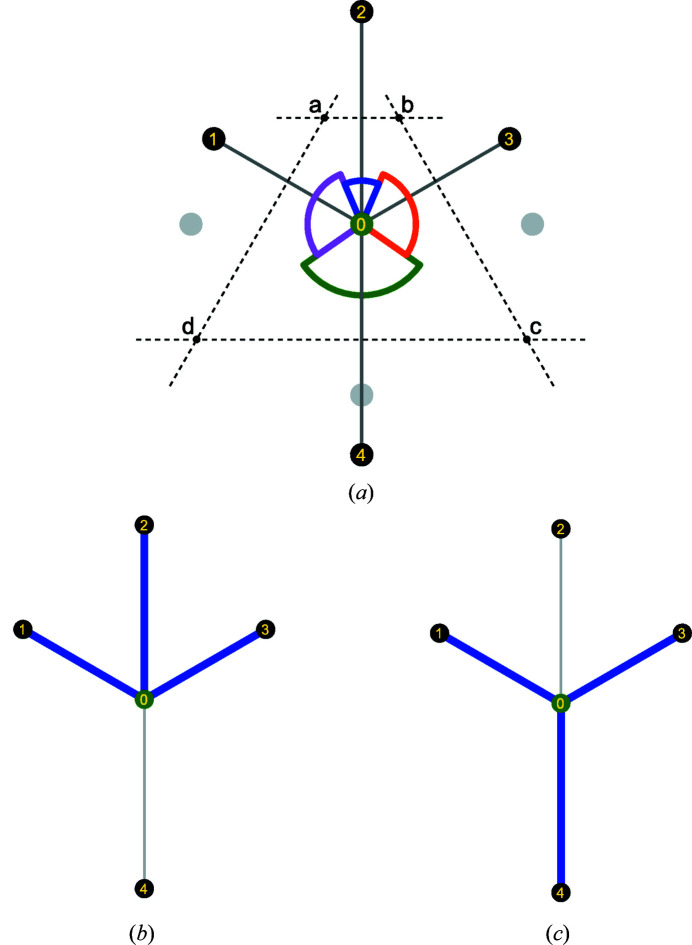
Illustration in two dimensions of two sets of neighbors having the same coordination number. (*a*) Local environment of atom 0. Normalized distances to neighbors 1, 2, 3 and 4 are 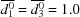, 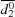 = 1.15 and 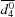 = 1.35. Normalized angles to neighbors 1, 2, 3 and 4 are 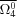 = 1.0, 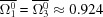 and 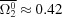. (*b*) Set of neighbors (1, 2 and 3) of atom 0 with *N* = 3. This set of neighbors is obtained with *e.g.* κ = 1.25 and γ = 0.3 cut-offs. (*c*) Another set of neighbors (1, 3 and 4) of atom 0 with *N* = 3. This set of neighbors is obtained with *e.g.* κ = 1.4 and γ = 0.5 cut-offs.

The distance and angle parameters defined in Section 2.2 are very sensitive parameters for the determination of the neighbors of a given atom. Indeed, a very slight change in one of the parameters can change the atoms considered as neighbors and hence the coordination. Each neighbor set of atom *A* with coordination *N* is denoted by Ξ_*N*, *j*_(*A*). The *j* index comes from the fact that two different neighbor sets can have the same coordination *N*. A two-dimensional example of such a case is illustrated in Fig. 10 ▸ in which two sets of distance and angle cut-off parameters result in two different neighbor sets of the same coordination.

**Figure 11:**
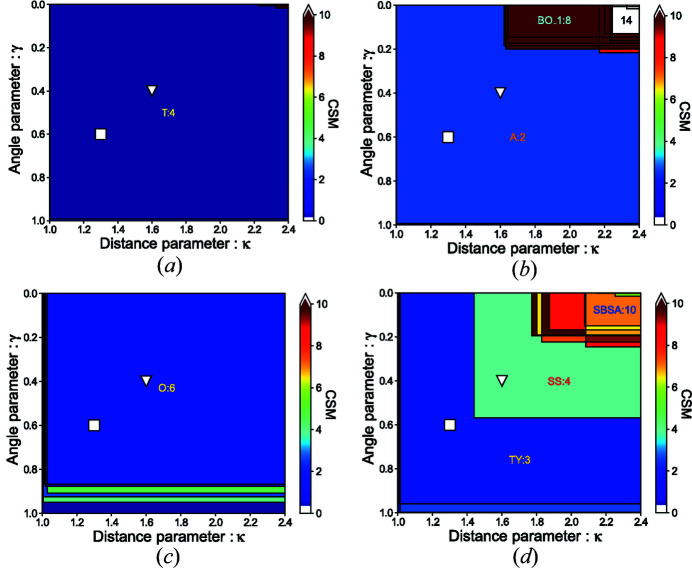
Examples of distance/angle parameters maps for Si and O in α-SiO_2_ (Materials Project id: mp-7000) and Cr and Te in Cr_2_Te_4_O_11_ (Materials Project id: mp-540537): (*a*) Si site in α-SiO_2_, (*b*) O site in α-SiO_2_, (*c*) Cr site in Cr_2_Te_4_O_11_ and (*d*) Te site in Cr_2_Te_4_O_11_. Each neighbor set corresponds to a region in which any distance/angle parameters combination result in the same set. The color level of each region gives an indication of the CSM value of the model polyhedron to which the corresponding neighbor set resembles the most (*i.e.* for which the CSM is the lowest). For the larger regions, this model polyhedron is indicated by its symbol. The square and triangle symbols correspond to fixed distance and angle parameters respectively of 1.3/0.6 and 1.6/0.4, showing a clear ambiguity for the Te site in Cr_2_Te_4_O_11_ (see Section 2.6 on how to clarify such cases).

In order to ensure robustness with respect to the distance and angle cut-off parameters, the identification procedure is performed in two steps. First, all sets of neighbors Ξ_*N*, *j*_(*A*) are obtained for all possible distance/angle parameters in the Voronoï analysis. For each neighbor set, CSMs are computed with respect to each model polyhedron of the same coordination. This can be represented by a *map* of distance/angle parameters with regions defined for each neighbor set (see Fig. 11 ▸ for examples of such maps for Si and O sites in SiO_2_ as well as for Cr and Te sites in Cr_2_Te_4_O_11_). The second step allows one to test the sensitivity of the distance/angle parameters by means of *strategies* (see Section 2.6). While for the three first cases in Fig. 11 ▸, the ‘correct’ environment is reasonably clear by just looking at the figure (assigning tetrahedral (T:4), angular (A:2) and octahedral (O:6) environments, respectively, to Si in SiO_2_, O in SiO_2_ and Cr in Cr_2_Te_4_O_11_), the situation is more complex and the identification is not so evident for Te in Cr_2_Te_4_O_11_. In this case, the environment could be seen as an *intermediate* between two different environments. The use of *strategies* can clarify such ambiguous cases.

The neighbors in each set, the CSMs for each model polyhedron in each set, and other data related to each neighbor set are stored in a so-called *StructureEnvironments* (see also Section 3) or SE (hereafter also symbolized by Φ_*A*_ for atom *A*) object. As exemplified in Fig. 11 ▸, this SE is not very useful as such as it contains a lot of information that is difficult to interpret directly. In the second step presented below, *strategies* are used to analyze the SE and extract usable and valuable information from the SE.

### 2.6. Strategies   

For the final step of the identification procedure, *strategies* are used to reliably analyze the SE object and extract a usable and meaningful result. Reliability refers to the robustness of our algorithm in which the sensitivity of the identification to the distance/angle parameters is tested and challenged. Hence, the local environments can be interpreted as one unique environment or as an intermediate between two (or more) coordination environments, each of which being attributed a fraction or percentage. Different strategies can be used depending on the goals, needs and constraints required by the user. This flexibility provided by the strategies is one of the strengths of our identification procedure. For visualization purposes, a strategy resulting in the identification of a single coordination environment for each site has to be used while reviewing the most commonly observed environments can be done with a strategy allowing for multiple environments for the same site. One can also favor specific or larger/smaller environments depending on the project. In the following, two strategies are developed further.

#### 2.6.1. Fixed distance/angle cut-offs strategy   

**Figure 12:**
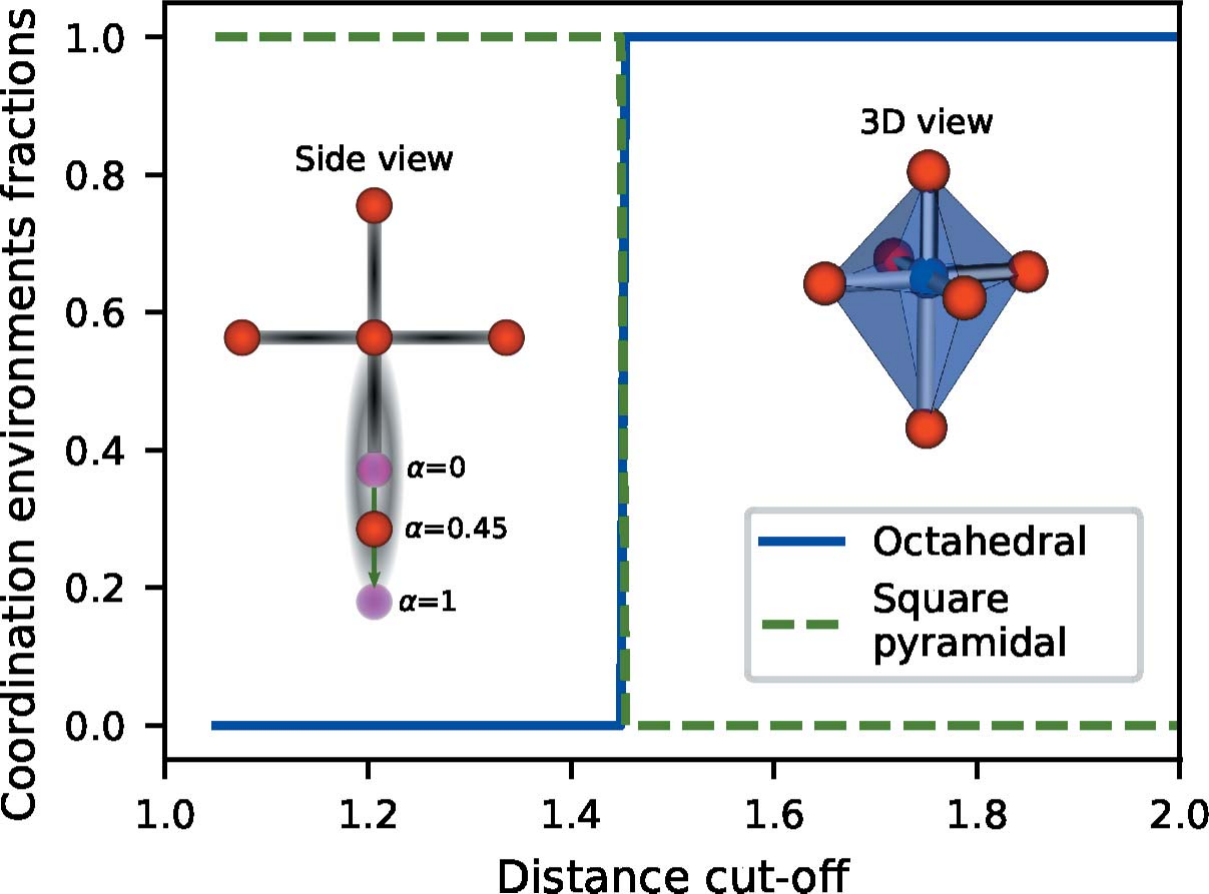
Coordination environments for a distorted octahedron in which the bottom atom is at distance 1.45 times larger than the other five neighbors. When the distance cut-off is lower than 1.45, the bottom atom is not considered as a neighbor and the environment is identified as a square pyramid. When the distance cut-off is larger than 1.45, the bottom atom is taken into account and the environment is identified as an octahedron.

**Figure 13:**
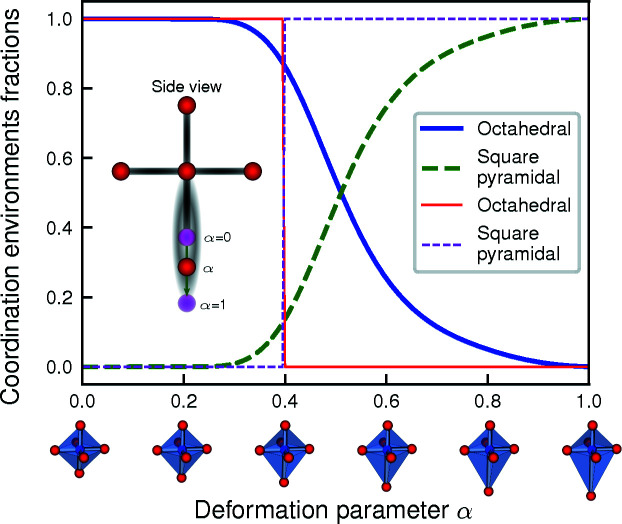
Smooth distortion from octahedral to square-pyramidal environment by moving away the bottom atom. The deformation parameter α = 0 corresponds to the perfect octahedron while for α = 1, the bottom atom has been moved to a distance that is twice that of the distance to the other neighbors. The thin lines gives the fractions of octahedral and square-pyramidal environments obtained with the *SimplestChemenvStrategy* (with a distance cut-off of 1.4) while the thick lines correspond to the fractions obtained with the *MultiWeightsChemenvStrategy*. Octahedral and square-pyramidal are respectively shown as solid and dashed lines.

The simplest way to identify the environment is to use fixed distance and angle cut-off parameters. In this *Simplest­Chemenv­Strategy*, the set of neighbors is thus unique and the environment is identified as the one for which the CSM is the lowest. The advantage of such a simple procedure is that it makes it possible to describe a local environment by its unique corresponding model environment, which is easier to use for visualization purposes. However, some (distorted or very distorted) local environments can be considered to be an intermediate between two or more model coordination polyhedra. In such cases, this strategy will simply ‘choose’ one environment, depending on the distance and angle parameters. As a simple illustration, Fig. 12 ▸ shows the sudden switch from the square-pyramidal environment to the octahedral environment when the distance cut-off is increased. Similarly, for fixed distance and angle cut-offs, when an octahedron is smoothly distorted by moving away one of the atoms, the resulting environment from this simplest strategy changes abruptly from octahedral to square-pyramidal as shown in Fig. 13 ▸ (thin lines correspond to the *SimplestChemenvStrategy*). It is thus very sensitive to small changes in the positions of the atoms. Nevertheless, with decent distance and angle parameters (*e.g.* κ = 1.4 and γ = 0.3), the identified environment is reasonably correct in about 85% of the cases.

**Figure 14:**
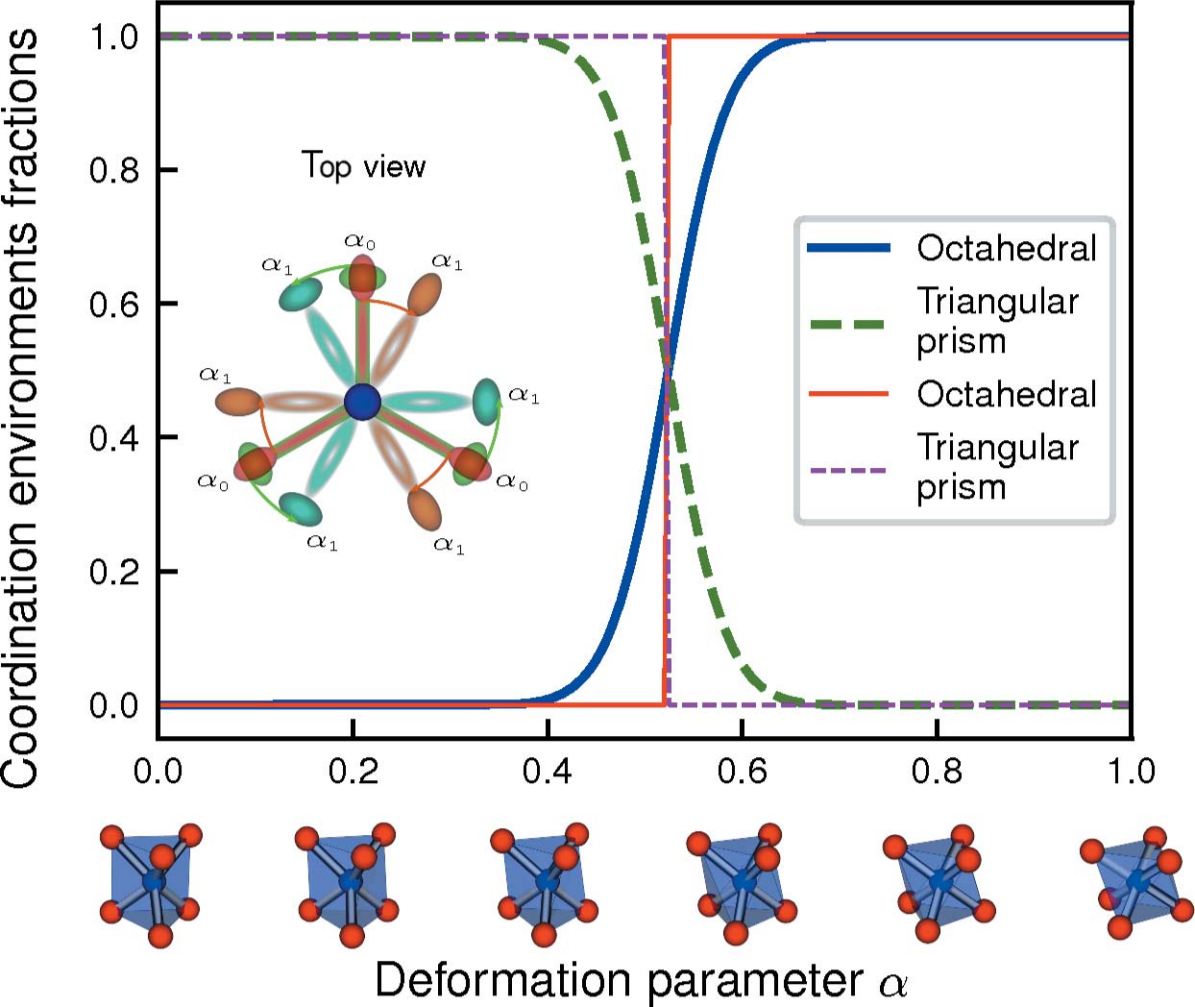
Smooth distortion from triangular prismatic to octahedral environment by twisting the triangular prism around the principal axis. The deformation parameter α = 0 corresponds to the perfect trigonal prism while for α = 1, the upper (red → orange) and lower (green → cyan) triangles have been rotated respectively clockwise and counterclockwise by 30°, corresponding to an octahedron. The thin lines gives the fractions of triangular prismatic and octahedral environments obtained with the *SimplestChemenvStrategy* while the thick lines correspond to the fractions obtained with the *MultiWeightsChemenvStrategy*. Octahedral and triangular prismatic are respectively shown as solid and dashed lines.

Another illustration of this strategy is shown in Fig. 14 ▸ in which a triangular prism is smoothly distorted towards an octahedron by rotating the upper and lower triangular planes in opposite directions (thin lines correspond to the *SimplestChemenvStrategy*). In this case, the number of neighbors remains the same while the actual identified environment switches abruptly from triangular prismatic to octahedral when the CSM of latter becomes smaller than that of the former. Once again, the sensitivity with respect to small changes in the positions of the atoms is critical in this strategy.

#### 2.6.2. Strategy based on multiple weights   

A second strategy is developed hereafter, in which special care has been taken to remove the artificial abrupt transitions observed with the *SimplestChemenvStrategy*. The idea is to smooth these transitions using a combination of smooth step functions. A given local environment can thus be identified either as one unique coordination environment if distortions are small, or as a *mix* of two or more environments for larger distortions. In practice, the local environment is described as a list of environments, each being assigned a *fraction* or *percentage*.

The percentage or fraction *f*_∊_(*A*) of a given model coordination environment ∊ depends on the results (CSMs, Voronoï parameters, …) for each possible set of neighbors contained in Φ_*A*_.
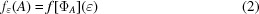
The procedure used to get the fraction of a model polyhedron ∊ for a given local environment is then obtained as the product of two terms. Suppose ∊ occurs in a given neighbor set Ξ. The first term results from the relative weight of the neighbor set (as compared to the other neighbor sets) displaying model environment ∊. The second term comes from the relative weight of the model polyhedron ∊ within that specific neighbor set.
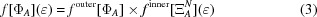
In the following, the first term is referred to as the *outer weight* (*i.e.* the weight that depends on other so-called *outer* neighbor sets) and the second term is referred to as the *inner weight* (*i.e.* the weight *inside* a specific neighbor set).

**Inner weight**. For a given neighbor set 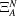 of atom *A* in a given coordination *N*, the relative weight (and hence fraction) of each model polyhedron is not straightforward. Let Θ^*N*^ be the set of *K* model environments with coordination *N*: 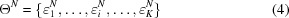
For example, the set Θ^6^ of six-coordinated model polyhedra [as reported by Hartshorn *et al.* (2007 ▸) and Lima-de Faria *et al.* (1990 ▸) and implemented in the *ChemEnv* package] is composed of the octahedron (symbolized O:6), the trigonal prism (symbolized T:6) and the pentagonal pyramid (symbolized PP:6).

**Figure 15:**
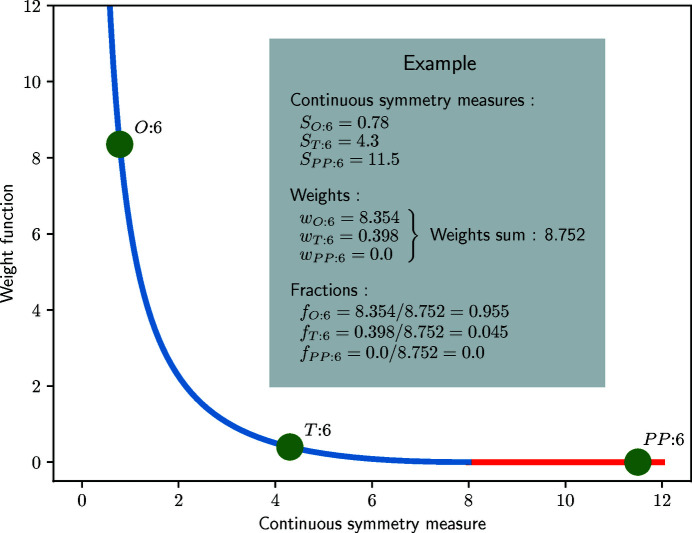
Weight function for the *inner* weight of model polyhedra. In this example, *S*_max_ is set to 8.0, so that the weight of any model polyhedron with a CSM larger than 8.0 is zero.

For each model polyhedron 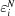, the CSM 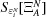 with respect to the local environment 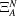 is used to assign a weight to each model polyhedron thanks to the use of an adequately shaped function. Model environments with a lower CSM (*i.e.* more similar to the local environment) are assigned a larger weight. In particular, if one of the model environments has a CSM of 0.0 (*i.e.* the local environment is perfect), its weight should be infinite so that it is the only model environment identified. The function should also allow for the assignment of a zero weight to a model polyhedron for which the CSM is larger than a given maximum value *S* ^max^. One example of such a function is the ‘modified’ inverse function defined in equation (5) and shown in Fig. 15 ▸.
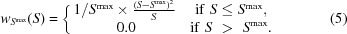
in which the numerator (*S* − *S* ^max^)^2^ ensures the continuity at *S* = *S* ^max^ while the prefactor 1/*S* ^max^ arises from the normalization of the [0, *S* ^max^] to [0, 1].

Fractions of each model environment 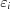 are then obtained from these weights using equation (6):
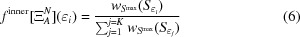
A small example is also given in Fig. 15 ▸ in which CSMs for a fictitious six-coordinated case are provided.

When the coordination is clearly defined (*i.e.* when only one neighbor set is identified using the procedure outlined in 2.5), the fractions of each model polyhedron are solely determined by this inner weight. On the other hand, when different neighbor sets are identified, an additional complexity arises from the fact that smaller environments usually tend to be more easily recognized as similar (*i.e.* having smaller CSMs). The extreme case is the single neighbor which is always assigned a CSM of zero. For cases in which more than one neighbor set is present, the outer weight is used to determine the relative predominance of each of the neighbor sets (and hence their corresponding model polyhedron).

**Outer weight**. The *outer weight* or *neighbor set weight* refers to the weight of a given neighbor set with respect to the other neighbor sets. This outer weight is defined as a product of several ‘partial weights’ (the definition being general enough to allow for flexibility in the choice of the weights): 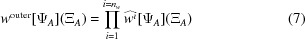
in which *n*_*w*_ is the number of partial weights used.

Some of the partial neighbor set weights compare the CSMs of this neighbor set with the ones for the other neighbor sets. The simplest approach is to take the smallest CSM for each of the neighbor sets. In practice, to ensure continuity, an *effective* CSM is defined. The effective CSM of a given neighbor set 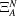, denoted 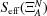, is obtained from a weighted average using the ‘modified’ inverse function defined in equation (5)
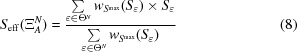
in which 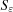 is a short form for 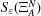, *i.e.* the CSM of the neighbor set with respect to the perfect environment 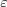.

**Partial weights**. In the following, the partial weights used in the ‘default’ multi-weights strategy [used in a previous publication (Waroquiers *et al.*, 2017 ▸)] are described. The strategy with these default parameters is easily obtained with the following class method (see examples in the tutorials provided in the supplementary material):
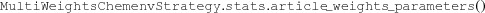



Other weights have also been implemented in the *ChemEnv* package in *pymatgen*.

**Figure 16:**
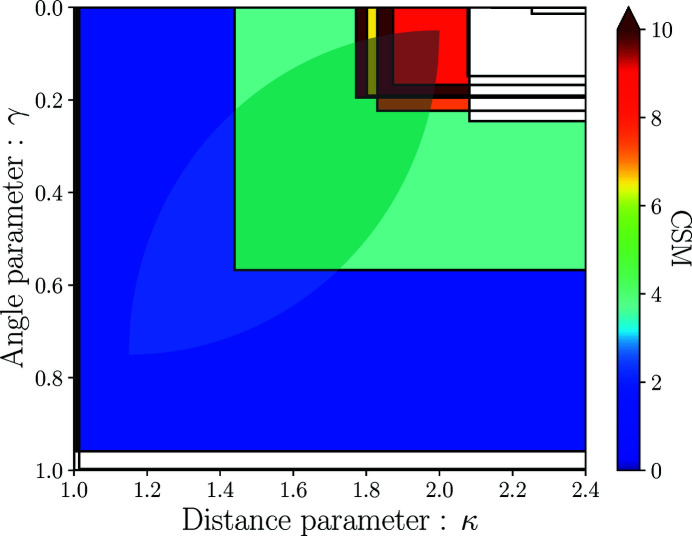
Schematic of the distance–angle area weight. The shaded area is used to determine which neighbor sets are considered. If the region of a given neighbor set is crossing the shaded area, the set is assigned a ‘distance–angle area’ weight of 1.0. In the opposite case, the set is assigned a weight of 0.0 (white regions).

**‘Distance–angle area’ weight**. The idea is to restrict the neighbor sets to those originating from a specific range of values for the distance and angle cutoffs. For example, one might only consider distance cutoffs between 1.2 and 1.8. One might also consider that the Voronoï angle towards a neighbor should always be between 0.3 and 0.8. In practice, a special *area* of distance–angle parameters is defined such as the one shown in Fig. 16 ▸. Indeed, there is not much sense to allow for neighbors with a small angle parameter and a small distance parameter or with a large angle parameter and a large distance parameter. If the region of a given neighbor set (as defined in Section 2.5) is crossing the above-mentioned area, the weight of this neighbor set is 1.0 (indicated in white on Fig. 16 ▸), otherwise it is set to 0.0. An extension of this weight could be to ensure it is continuous.

**‘Self CSM’ weight**

**Figure 17:**
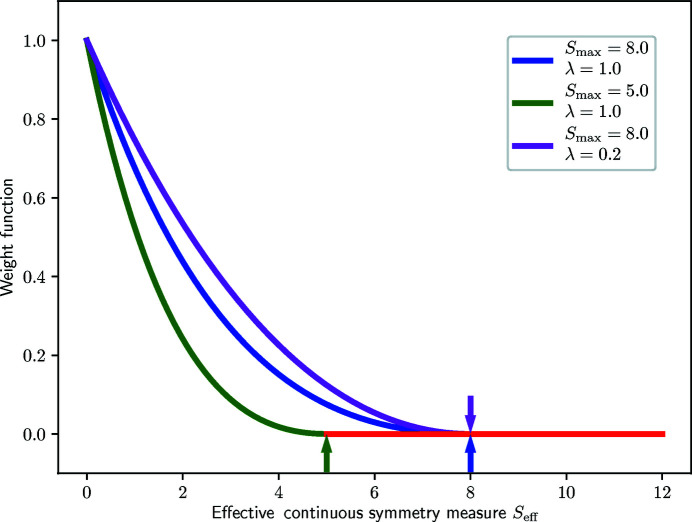
Weight function for the Self-CSM *outer* weight of neighbor sets as defined in equation (9). The default parameters for this weight are shown as blue while the green and purple curves illustrate other parameters. Arrows indicate thresholds above which values (*i.e. S*_max_) of the effective CSM *S*_eff_ each of the weight functions are set to zero.

This weight makes use of the effective CSM *S*_eff_ of each neighbor set defined in equation (8). Each neighbor set is assigned a weight depending on the value of this effective *S*_eff_. The idea is to disfavor neighbor sets that are *globally* more distorted than others. One example function used to estimate this weight is defined in equation (9) and shown in Fig. 17 ▸.
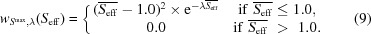
where 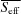 is the normalized effective CSM defined as 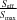.

**Delta CSM’ weight**. The goal of this neighbor set weight is to reduce the importance of a given neighbor set 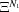 if another neighbor set 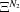 of larger coordination number *N*_2_ > *N*_1_ is present and not too distorted with respect to the first one. In practice, this weight depends on the difference Δ*S*_eff_ between the effective CSMs [as defined in equation (8)] of the neighbor sets 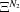 and 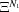: 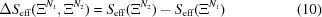
The Delta CSM weight is defined as: 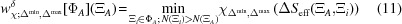
in which χ is a sigmoid-like function (*e.g.* a smooth step or smoother step function), *N*(Ξ) is the coordination of neighbor set Ξ and Δ_min_, Δ_max_ are the edges used in the χ function.

**Figure 18:**
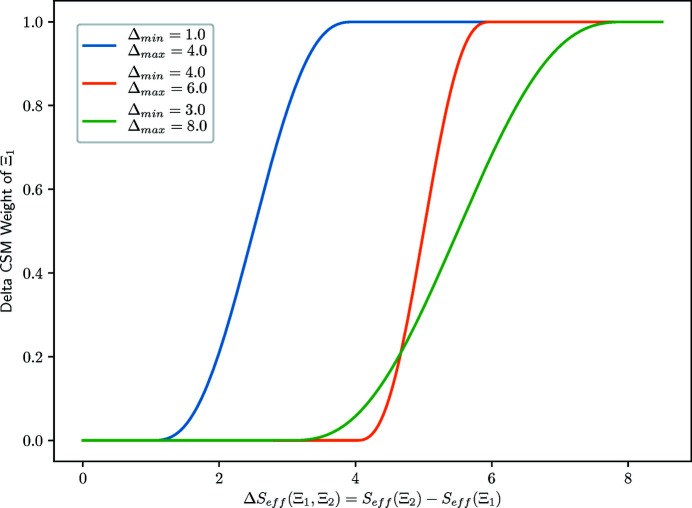
Smoother step function used in the Delta CSM weight. The ‘Delta CSM’ weight assigned to the Ξ_1_ neighbor set is equal to 0.0 if the difference Δ*S*_eff_(Ξ_1_, Ξ_2_) between the effective CSM *S*_eff_(Ξ_2_) of the Ξ_2_ neighbor set and its own effective CSM *S*_eff_(Ξ_1_) is lower than Δ_min_. If the difference Δ*S*_eff_(Ξ_1_, Ξ_2_) is larger than Δ_max_, the Ξ_1_ set is assigned a weight of 1.0. The smoother step function is used between these two extremes. The Δ_min_ and Δ_max_ values can be changed if needed and examples of smoother step functions for different values are shown.

An example of a χ function is the smoother step function shown in Fig. 18 ▸ and defined as: 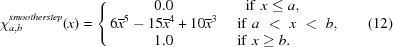
in which 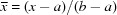 is the scaled value of *x* mapping the [*a,b*] interval to the [0, 1] interval.

**Figure 19:**
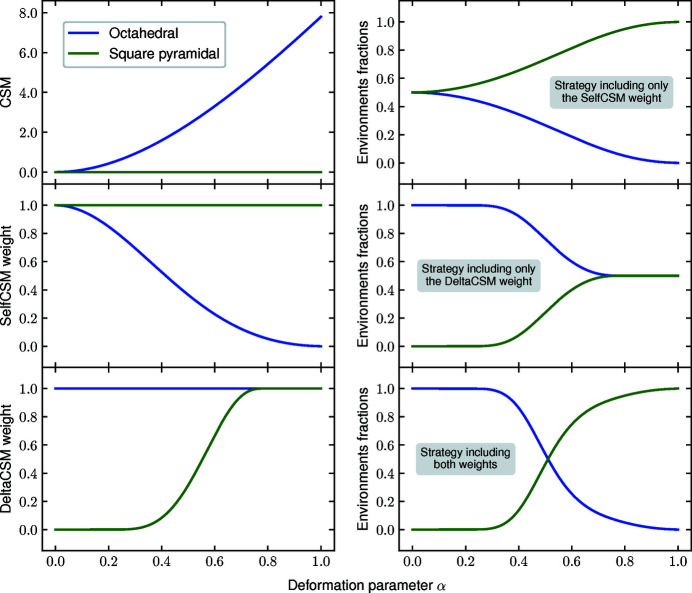
Choice of partial weights: comparison and combination of Self CSM and Delta CSM weights in the case of the smooth distortion from octahedral to square-pyramidal environment. Curves in blue (green) correspond to the octahedral (square-pyramidal) environment. See text for details.

**Choice of partial weights**. The default list of outer weights consists of the three above-mentioned partial weights. As an example and in particular to illustrate the need to use both the Self CSM weight and the Delta CSM weight, Fig. 19 ▸ shows the fractions of environments obtained for different choices of weights in the case of the smooth distortion from octahedral to square-pyramidal environment (see Fig. 13 ▸).

The upper left panel shows the CSM of the octahedral (increasing with the distortion) and square-pyramidal (always equal to 0.0). The middle left and lower left panels show the Self CSM and Delta CSM weights for both environments. The Self CSM weight for the square-pyramidal environment is always 1.0 as its CSM is always 0.0. Conversely, the Delta CSM weight for the octahedral environment is always 1.0 as there is no larger neighbor set to be compared to. As shown in the upper right panel, when the sole Self CSM weight is included, the fractions obtained are 50% octahedral and 50% square-pyramidal when no or little distortion is applied (while one would expect to have 100% octahedral and 0% square-pyramidal). Indeed, for both environments, the value of the CSM is 0.0 and hence the Self CSM weight is 1.0. At variance, the middle right panel illustrates the fractions obtained when the sole Delta CSM weight is included. In that case, for large distortions, the fractions obtained are also 50% for each environment. Indeed, when the distortion is large, the Delta CSM weight for the square-pyramidal environment reaches 1.0 as the larger environment is too distorted to disfavor the square-pyramidal environment. The lower right panel illustrates the case when both the Self CSM and Delta CSM weights are included.

## 3. Description of the package   

**Figure 20:**
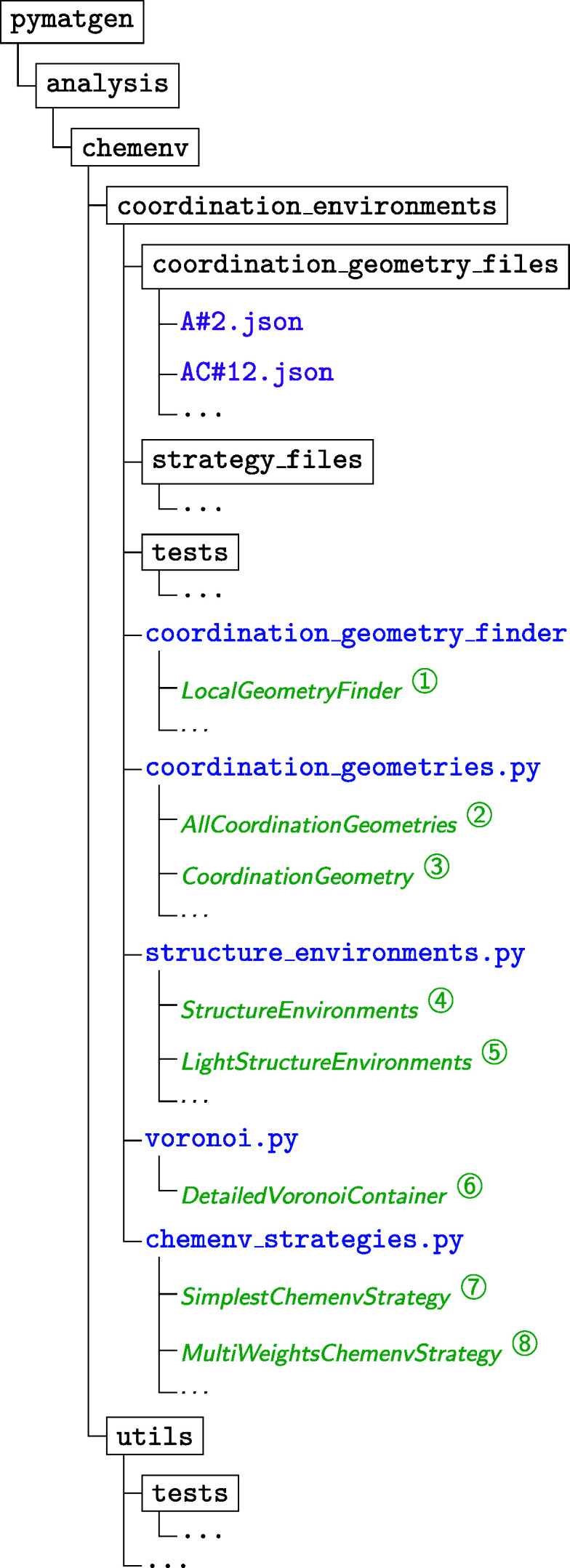
Organization of the *ChemEnv* package. Directories are indicated in black and surrounded by a rectangle. Files are indicated in typewriter (blue for python files, purple for other files). The most important python objects are indicated in italic (green). See text for more information.

The *ChemEnv* module is written in Python and can be found in the *pymatgen* package (Ong *et al.*, 2013 ▸) as part of the 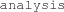 submodule. The organization of the package is shown diagrammatically in Fig. 20 ▸. The description of each of the objects referenced as circled numbers in this figure is given hereafter:


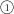
 *LocalGeometryFinder*

Main class used to identify the local environments in a structure.


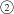
 *AllCoordinationGeometries*

Class containing the list of all the available model coordination geometries (as CoordinationGeometry objects, see 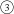


*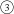 CoordinationGeometry*

Generic class for the description of all the model coordination geometries. An instance of this class is created for each model environment (from the json files stored in the 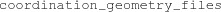 directory). It contains information about its perfect coordinates as well as its edges and faces, name(s), symbol(s), technical details for the identification procedure, ….


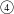
 *StructureEnvironments*

Class containing the information (CSMs, neighbors, …) on all possible neighbor sets for all sites in the structure as introduced in Section 2.5. This object is meant to be post-processed with a strategy in order to get relevant and usable data about the local environments of the structure.


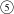
 *LightStructureEnvironments*

Class containing the processed data from the *StructureEnvironments* class using one strategy. This object lists the environment(s) and their corresponding fractions (in case of a strategy allowing for mixtures of environments) for each site of the structure.


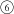
 *DetailedVoronoiContainer*

Class containing the information on the Voronoï analysis (see Section 2.2) performed at the beginning of the identification procedure in order to define the different possible neighbor sets.


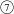
 *SimplestChemenvStrategy*

Class used to apply the fixed distance/angle cutoff strategy introduced in Section 2.6.1.


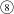
 *MultiWeightsChemenvStrategy*

Class used to apply the strategy based on multiple weights as introduced in Section 2.6.2.

**Figure 21:**
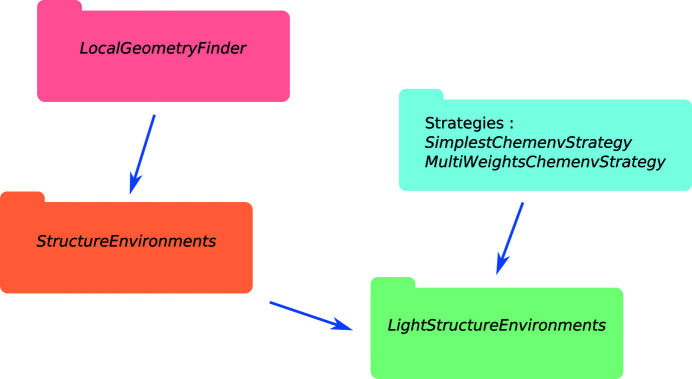
Main objects of the *ChemEnv* package.

The most relevant objects needed for the user of *ChemEnv* package are illustrated in Fig. 21 ▸.

The *LocalGeometryFinder* object is the main class used to initialize and set up the structure as well as to compute the *StructureEnvironments* object (containing the raw coordination environments data as introduced in Section 2.5). Combining this *StructureEnvironments* object with a strategy (*e.g. SimplestChemenvStrategy* or *MultiWeightsChemenv­Strategy*) leads to the *LightStructureEnvironments* object. This latter object contains the usable information about the environments in a structure, *i.e.* the environment or *mix* of environments (with their corresponding fractions) that is identified for each site.

## 4. Interactive web app   

An interactive web app has been developed to improve accessibility of the *ChemEnv* algorithms as part of the Materials Projects Crystal Toolkit platform. While the Python interface is intuitive and well documented, not all scientists are Python users, and the web app enables use of *ChemEnv* by any user without installing custom software. The web app supports uploading of any file format supported by the *pymatgen* code, including Crystallographic Information Format (CIF). Alternatively, structures can be loaded directly from the Materials Project database containing more than 100 000 inorganic materials.

The web app is designed to offer one-to-one equivalent functionality to *ChemEnv* by directly calling the corresponding *pymatgen* interface, specifically using the *LightStructureEnvironments* and *SimplestChemenvStrategy*, and allowing the user full interactive control over the distance and angle cut-offs. Each symmetrically distinct chemical environment is shown in 3D using a custom atomic visualizer, along with Wyckoff label, IUPAC symbol, CSM, and human-readable environment label. Oxidation states will be used in the analysis if atoms are appropriately annotated in the uploaded file or, if these are not supplied, oxidation states can be guessed on-the-fly using *pymatgen*’s bond valence analysis algorithms. It will be hosted by the Materials Project, and is available at http://crystaltoolkit.org.

## 5. Conclusion   

We have developed a tool that can analyze coordination or local environments of large numbers of crystal structures in a fast and robust manner. The analysis of the neighboring atoms relies on a modified Voronoï approach based on a grid of distance. From this grid of different distance and angle cutoffs, the coordination environments are determined with the help of a similarity metric to the shape of ideal polyhedra. Two different strategies are implemented to arrive at the final assignment of the coordination environments. One of these strategies is especially robust against small distortions of the crystal structures making the algorithm particularly useful for automatic, unsupervised, local environment assignment. This new tool can be used as part of the open-source Python library (*pymatgen*) and within an interactive web app available on http://crystaltoolkit.org through the Materials Project.

## 6. Supporting information   

A tutorial for the *ChemEnv* package, in both pdf and jupyter-notebook format, is available in the supporting information. The list of all environments as well as some details about the implementation are also available in the supporting information. 

## Supplementary Material

List of all environments and some details about the implementation. DOI: 10.1107/S2052520620007994/lo5066sup3.pdf

Tutorial for ChemEnv package. DOI: 10.1107/S2052520620007994/lo5066sup1.pdf

Tutorial for the ChemEnv in jupyter-notebook format. DOI: 10.1107/S2052520620007994/lo5066sup2.txt
