# Supplementary material for: ChemEnv: a fast and robust coordination environment identification tool
Source: Acta Crystallogr B Struct Sci Cryst Eng Mater. 2020 Jul 21;76(Pt 4):683–95. doi: 10.1107/S2052520620007994 (PMC7412753; doi:10.1107/S2052520620007994)
Supplement: Supplementary file 1 [file b-76-00683-sup3.pdf]

Acta Crystallographica Section A
Foundations of Crystallography
ISSN 0108-7673

Supporting Information for "*ChemEnv* : A fast and robust coordination environment identification tool"

David Waroquiers,[a] Janine George,[a] Matthew Horton,[b,c] Stephan Schenk,[d] Kristin A. Persson,[b,c] Gian-Marco Rignanese,[a] Xavier Gonze[a,e] and Geoffroy Hautier [a]*

[a]Institute of Condensed Matter and Nanosciences, Université catholique de Louvain, Chemin des Étoiles 8, 1348 Louvain-la-Neuve, Belgium, [b]Energy Technologies Area, Lawrence Berkeley National Laboratory, Berkeley, CA 94720, USA, [c]Department of Materials Science and Engineering, University of California, Berkeley, CA 94720, USA, [d]BASF SE, Digitalization of R&D, Carl-Bosch-Str. 38, 67056 Ludwigshafen, Germany, and [e]Skolkovo Institute of Science and Technology, Skolkovo Innovation Center, Nobel St. 3, Moscow, 143026, Russia. Correspondence e-mail: geoffroy.hautier@uclouvain.be

© 0000 International Union of Crystallography
Printed in Singapore – all rights reserved

This supplementary information describes the different coordination environments identified by ChemEnv and provides technical details about the identification procedure.

1. Model coordination environments and separation planes

The following lists the model coordination environments for each coordination number. For each model coordination environment, the symbol used in *ChemEnv*, a descriptive name, the coordinates of the points, the IUCr and IUPAC symbols as well as technical details about the algorithm used for the identification are provided.

Coordination 1

- **S:1 → Single neighbor**
  IUCr symbol : [1l]
  IUPAC symbol : None
  Points :

A	0.0000	0.0000	1.0000

  Explicit permutations algorithm

Coordination 2

- **L:2 → Linear**
  IUCr symbol : [2l]
  IUPAC symbol : L-2
  Points :

A	0.0000	0.0000	1.0000
B	0.0000	0.0000	−1.0000

  Explicit permutations algorithm

- **A:2 → Angular**
  IUCr symbol : [2n]
  IUPAC symbol : A-2
  Points :

A	1.0000	0.0000	0.0000
B	−0.5000	0.8660	0.0000

  Explicit permutations algorithm

Coordination 3

- **TL:3 → Trigonal plane**
  IUCr symbol : [3l]
  IUPAC symbol : TP-3
  Points :

A	0.0000	1.0000	0.0000
B	0.8660	−0.5000	0.0000
C	−0.8660	−0.5000	0.0000

  Explicit permutations algorithm

- **TY:3 → Triangular non-coplanar**
  IUCr symbol : [3n]
  IUPAC symbol : TPY-3
  Points :

A	0.5774	−0.5774	−0.5774
B	−0.5774	0.5774	−0.5774
C	−0.5774	−0.5774	0.5774

  Explicit permutations algorithm

- **TS:3 → T-shaped**
  IUCr symbol : None
  IUPAC symbol : TS-3
  Points :

A	−1.0000	0.0000	0.0000
B	1.0000	0.0000	0.0000
C	0.0000	0.0000	1.0000

  Explicit permutations algorithm

Coordination 4

- **T:4 → Tetrahedron**
  IUCr symbol : [4t]
  IUPAC symbol : T-4
  Points :

A	0.5774	−0.5774	−0.5774
B	−0.5774	0.5774	−0.5774
C	−0.5774	−0.5774	0.5774
D	0.5774	0.5774	0.5774

  Explicit permutations algorithm

- **S:4 → Square plane**
  IUCr symbol : [4l]
  IUPAC symbol : SP-4
  Points :

A	1.0000	0.0000	0.0000
B	−1.0000	0.0000	0.0000
C	0.0000	1.0000	0.0000
D	0.0000	−1.0000	0.0000

  Explicit permutations algorithm

- **SY:4 → Square non-coplanar**
  IUCr symbol : [4n]
  IUPAC symbol : SPY-4
  Points :

A	0.9258	0.0000	0.3780
B	−0.9258	0.0000	0.3780
C	0.0000	0.9258	0.3780
D	0.0000	−0.9258	0.3780

  Explicit permutations algorithm

- **SS:4 → See-saw**
  IUCr symbol : None
  IUPAC symbol : SS-4
  Points :

A	1.0000	0.0000	0.0000
B	0.0000	0.8660	0.5000
C	0.0000	0.0000	−1.0000
D	−1.0000	0.0000	0.0000

  Explicit permutations algorithm

Coordination 5

- **PP:5 → Pentagonal plane**
  IUCr symbol : [5l]
  IUPAC symbol : PP-5
  Points :

A	1.0000	0.0000	0.0000
B	0.3090	0.9511	0.0000
C	−0.8090	0.5878	0.0000
D	−0.8090	−0.5878	0.0000
E	0.3090	−0.9511	0.0000

  Explicit permutations algorithm

- **S:5 → Square pyramid**
  IUCr symbol : [5y]
  IUPAC symbol : SPY-5
  Points :

A	1.0000	0.0000	0.0000
B	−1.0000	0.0000	0.0000
C	0.0000	1.0000	0.0000
D	0.0000	−1.0000	0.0000
E	0.0000	0.0000	1.0000

  Explicit permutations algorithm

- **T:5 → Trigonal bipyramid**
  IUCr symbol : [5by]
  IUPAC symbol : TBPY-5
  Points :

A	0.0000	1.0000	0.0000
B	0.8660	−0.5000	0.0000
C	−0.8660	−0.5000	0.0000
D	0.0000	0.0000	1.0000
E	0.0000	0.0000	−1.0000

  Explicit permutations algorithm

Coordination 6

- **O:6 → Octahedron**
  IUCr symbol : [6o]
  IUPAC symbol : OC-6
  Points :

A	0.0000	0.0000	1.0000
B	0.0000	0.0000	−1.0000
C	1.0000	0.0000	0.0000
D	−1.0000	0.0000	0.0000
E	0.0000	1.0000	0.0000
F	0.0000	−1.0000	0.0000

  Separation plane algorithms :
  ➔ E / ACBD / F
  ➔ ∅ / ACE / FBD

- **T:6 → Trigonal prism**
  IUCr symbol : [6p]
  IUPAC symbol : TPR-6
  Points :

A	−0.6547	−0.3780	0.6547
B	0.6547	−0.3780	0.6547
C	0.0000	0.7559	0.6547
D	−0.6547	−0.3780	−0.6547
E	0.6547	−0.3780	−0.6547
F	0.0000	0.7559	−0.6547

  Separation plane algorithms :
  ➔ ∅ / ABED / CF
  ➔ ∅ / ABC / DEF

- **PP:6 → Pentagonal pyramid**
IUCr symbol : None
IUPAC symbol : PPY-6
Points :

A	1.0000	0.0000	0.0000
B	0.3090	0.9511	0.0000
C	−0.8090	0.5878	0.0000
D	−0.8090	−0.5878	0.0000
E	0.3090	−0.9511	0.0000
F	0.0000	0.0000	1.0000

Separation plane algorithms :

➔ ∅ / ABCDE / F

➔ BC / AF / ED

Coordination 7

- **PB:7 → Pentagonal bipyramid**
IUCr symbol : [7by]
IUPAC symbol : PBPY-7
Points :

A	1.0000	0.0000	0.0000
B	0.3090	0.9511	0.0000
C	−0.8090	0.5878	0.0000
D	−0.8090	−0.5878	0.0000
E	0.3090	−0.9511	0.0000
F	0.0000	0.0000	1.0000
G	0.0000	0.0000	−1.0000

Separation plane algorithms :

➔ F / ABCDE / G

➔ DE / AFG / CB

- **ST:7 → Square-face capped trigonal prism**
IUCr symbol : [6p1c]
IUPAC symbol : TPRS-7
Points :

A	−0.6547	−0.3780	0.6547
B	0.6547	−0.3780	0.6547
C	0.0000	0.7559	0.6547
D	−0.6547	−0.3780	−0.6547
E	0.6547	−0.3780	−0.6547
F	0.0000	0.7559	−0.6547
G	0.0000	−1.0000	0.0000

Separation plane algorithms :

➔ G / ABED / CF

➔ AD / CFG / BE

- **ET:7 → End-trigonal-face capped trigonal prism**
IUCr symbol : None
IUPAC symbol : TPRT-7
Points :

A	−0.6547	−0.3780	0.6547
B	0.6547	−0.3780	0.6547
C	0.0000	0.7559	0.6547
D	−0.6547	−0.3780	−0.6547
E	0.6547	−0.3780	−0.6547
F	0.0000	0.7559	−0.6547
G	0.0000	0.0000	1.0000

Separation plane algorithms :

➔ G / ABC / DEF

➔ BE / AGD / CF

- **FO:7 → Face-capped octahedron**
IUCr symbol : None
IUPAC symbol : OCF-7
Points :

A	0.0000	0.0000	1.0000
B	0.0000	0.0000	−1.0000
C	1.0000	0.0000	0.0000
D	−1.0000	0.0000	0.0000
E	0.0000	1.0000	0.0000
F	0.0000	−1.0000	0.0000
G	0.5774	0.5774	0.5774

Separation plane algorithms :

➔ G / ACE / BDF

➔ D / AEBF / GC

Coordination 8

- **C:8 → Cube**
IUCr symbol : [8cb]
IUPAC symbol : CU-8
Points :

A	−0.5774	−0.5774	−0.5774
B	0.5774	−0.5774	−0.5774
C	−0.5774	0.5774	−0.5774
D	−0.5774	−0.5774	0.5774
E	−0.5774	0.5774	0.5774
F	0.5774	−0.5774	0.5774
G	0.5774	0.5774	−0.5774
H	0.5774	0.5774	0.5774

Separation plane algorithms :

➔ ∅ / ABGC / DFHE

➔ FD / ABHE / GC

- **SA:8 → Square antiprism**
  IUCr symbol : [8acb]
  IUPAC symbol : SAPR-8
  Points :

A	0.0000	0.8595	0.5111
B	0.0000	−0.8595	0.5111
C	0.8595	0.0000	0.5111
D	−0.8595	0.0000	0.5111
E	0.6078	0.6078	−0.5111
F	0.6078	−0.6078	−0.5111
G	−0.6078	0.6078	−0.5111
H	−0.6078	−0.6078	−0.5111

  Separation plane algorithms :
  ➔ ∅ / ACBD / EFHG
  ➔ E / ACFG / DBH

- **SBT:8 → Square-face bicapped trigonal prism**
  IUCr symbol : None
  IUPAC symbol : TPRS-8
  Points :

A	−0.6547	−0.3780	0.6547
B	0.6547	−0.3780	0.6547
C	0.0000	0.7559	0.6547
D	−0.6547	−0.3780	−0.6547
E	0.6547	−0.3780	−0.6547
F	0.0000	0.7559	−0.6547
G	0.8660	0.5000	0.0000
H	−0.8660	0.5000	0.0000

  Separation plane algorithms :
  ➔ ∅ / ABED / CGFH
  ➔ H / ACFD / BGE

- **TBT:8 → Triangular-face bicapped trigonal prism**
  IUCr symbol : [6p2c]
  IUPAC symbol : TPRT-8
  Points :

A	−0.6547	−0.3780	0.6547
B	0.6547	−0.3780	0.6547
C	0.0000	0.7559	0.6547
D	−0.6547	−0.3780	−0.6547
E	0.6547	−0.3780	−0.6547
F	0.0000	0.7559	−0.6547
G	0.0000	0.0000	1.0000
H	0.0000	0.0000	−1.0000

  Separation plane algorithm :
  ➔ AD / CFHG / BE

- **DD:8 → Dodecahedron with triangular faces**
  IUCr symbol : [8do]
  IUPAC symbol : DD-8
  Points :

A	−0.5000	0.0000	−0.7839
B	0.5000	0.0000	−0.7839
C	0.0000	−0.6446	−0.2056
D	0.0000	0.6446	−0.2056
E	−0.6446	0.0000	0.2056
F	0.6446	0.0000	0.2056
G	0.0000	−0.5000	0.7839
H	0.0000	0.5000	0.7839

  Separation plane algorithm :
  ➔ CG / ABFE / DH

- **DDPN:8 → Dodecahedron with triangular faces - p2345 plane normalized**
  IUCr symbol : None
  IUPAC symbol : None
  Points :

A	−0.5000	0.0000	−0.7839
B	0.5000	0.0000	−0.7839
C	0.0000	−0.9068	−0.2056
D	0.0000	0.9068	−0.2056
E	−0.9068	0.0000	0.2056
F	0.9068	0.0000	0.2056
G	0.0000	−0.5000	0.7839
H	0.0000	0.5000	0.7839

  Separation plane algorithm :
  ➔ CG / ABFE / DH

- **HB:8 → Hexagonal bipyramid**
  IUCr symbol : [8by]
  IUPAC symbol : HBPY-8
  Points :

A	1.0000	0.0000	0.0000
B	0.5000	0.8660	0.0000
C	−0.5000	0.8660	0.0000
D	−1.0000	0.0000	0.0000
E	−0.5000	−0.8660	0.0000
F	0.5000	−0.8660	0.0000
G	0.0000	0.0000	1.0000
H	0.0000	0.0000	−1.0000

  Separation plane algorithms :
  ➔ G / ABCDEF / H
  ➔ FE / AHDG / BC

- **BO_1:8 → Bicapped octahedron (opposed cap faces)**
  IUCr symbol : None
  IUPAC symbol : OCT-8
  Points :

A	0.0000	0.0000	1.0000
B	0.0000	0.0000	−1.0000
C	1.0000	0.0000	0.0000
D	−1.0000	0.0000	0.0000
E	0.0000	1.0000	0.0000
F	0.0000	−1.0000	0.0000
G	0.5774	0.5774	0.5774
H	−0.5774	−0.5774	−0.5774

  Separation plane algorithms :
  ➔ AD / EGFH / CB
  ➔ FH / ACBD / GE

- **BO_2:8 → Bicapped octahedron (cap faces with one atom in common)**
  IUCr symbol : None
  IUPAC symbol : OCT-8
  Points :

A	0.0000	0.0000	1.0000
B	0.0000	0.0000	−1.0000
C	1.0000	0.0000	0.0000
D	−1.0000	0.0000	0.0000
E	0.0000	1.0000	0.0000
F	0.0000	−1.0000	0.0000
G	0.5774	0.5774	0.5774
H	0.5774	−0.5774	−0.5774

  Separation plane algorithms :
  ➔ BE / CGDH / FA
  ➔ AG / CEDF / HB
  ➔ D / AEBF / CGH

- **BO_3:8 → Bicapped octahedron (cap faces with one edge in common)**
  IUCr symbol : None
  IUPAC symbol : OCT-8
  Points :

A	0.0000	0.0000	1.0000
B	0.0000	0.0000	−1.0000
C	1.0000	0.0000	0.0000
D	−1.0000	0.0000	0.0000
E	0.0000	1.0000	0.0000
F	0.0000	−1.0000	0.0000
G	0.5774	−0.5774	0.5774
H	0.5774	−0.5774	−0.5774

  Separation plane algorithms :
  ➔ CE / AGHB / FD
  ➔ AG / CEDF / BH
  ➔ E / ACBD / GHF

Coordination 9

- **TC:9 → Triangular cupola**
  IUCr symbol : None
  IUPAC symbol : TCA-9
  Points :

A	1.0000	0.0000	0.0000
B	0.5000	0.8660	0.0000
C	−0.5000	0.8660	0.0000
D	−1.0000	0.0000	0.0000
E	−0.5000	−0.8660	0.0000
F	0.5000	−0.8660	0.0000
G	0.0000	0.5774	0.8165
H	−0.5000	−0.2887	0.8165
I	0.5000	−0.2887	0.8165

  Separation plane algorithms :
  ➔ ∅ / ABCDEF / GHI
  ➔ EF / ADHI / BCG
  ➔ BC / ADG / FEHI

- **TT_1:9 → Tricapped triangular prism (three square-face caps)**
  IUCr symbol : [6p3c]
  IUPAC symbol : TPRS-9
  Points :

A	−0.6547	−0.3780	0.6547
B	0.6547	−0.3780	0.6547
C	0.0000	0.7559	0.6547
D	−0.6547	−0.3780	−0.6547
E	0.6547	−0.3780	−0.6547
F	0.0000	0.7559	−0.6547
G	0.8660	0.5000	0.0000
H	−0.8660	0.5000	0.0000
I	0.0000	−1.0000	0.0000

  Separation plane algorithms :
  ➔ ABC / GHI / DEF
  ➔ AID / BEH / CGF
  ➔ I / ABED / CGFH

- **TT_2:9 → Tricapped triangular prism (two square-face caps and one triangular-face cap)**
  IUCr symbol : [6p3c]
  IUPAC symbol : TPRS-9
  Points :

A	−0.6547	−0.3780	0.6547
B	0.6547	−0.3780	0.6547
C	0.0000	0.7559	0.6547
D	−0.6547	−0.3780	−0.6547
E	0.6547	−0.3780	−0.6547
F	0.0000	0.7559	−0.6547
G	0.8660	0.5000	0.0000
H	−0.8660	0.5000	0.0000
I	0.0000	0.0000	1.0000

Separation plane algorithms :

➔ AD / HIBE / CGF

➔ GEB / CFI / HDA

- **TT_3:9 → Tricapped triangular prism (one square-face cap and two triangular-face caps)**
IUCr symbol : [6p3c]
IUPAC symbol : TPRS-9
Points :

A	−0.6547	−0.3780	0.6547
B	0.6547	−0.3780	0.6547
C	0.0000	0.7559	0.6547
D	−0.6547	−0.3780	−0.6547
E	0.6547	−0.3780	−0.6547
F	0.0000	0.7559	−0.6547
G	0.0000	−1.0000	0.0000
H	0.0000	0.0000	−1.0000
I	0.0000	0.0000	1.0000

Separation plane algorithms :

➔ AD / IGHFC / BE

➔ CF / BEHI / AGD

- **HD:9 → Heptagonal dipyramid**
IUCr symbol : None
IUPAC symbol : HBPY-9
Points :

A	1.0000	0.0000	0.0000
B	0.6235	0.7818	0.0000
C	−0.2225	0.9749	0.0000
D	−0.9010	0.4339	0.0000
E	−0.9010	−0.4339	0.0000
F	−0.2225	−0.9749	0.0000
G	0.6235	−0.7818	0.0000
H	0.0000	0.0000	1.0000
I	0.0000	0.0000	−1.0000

Separation plane algorithm :

➔ H / ABCDEFG / I

- **TI:9 → Tridiminished icosahedron**
IUCr symbol : None
IUPAC symbol : None
Points :

A	0.0000	0.5257	0.8507
B	0.0000	0.5257	−0.8507
C	0.0000	−0.5257	−0.8507
D	0.5257	0.8507	0.0000
E	0.5257	−0.8507	0.0000
F	−0.5257	−0.8507	0.0000
G	0.8507	0.0000	0.5257
H	−0.8507	0.0000	0.5257
I	−0.8507	0.0000	−0.5257

Separation plane algorithms :

➔ GE / AFCD / HIB

➔ B / CDI / EGAHF

- **SMA:9 → Square-face monocapped antiprism**
IUCr symbol : None
IUPAC symbol : SAPRS-9
Points :

A	0.0000	0.8595	0.5111
B	0.0000	−0.8595	0.5111
C	0.8595	0.0000	0.5111
D	−0.8595	0.0000	0.5111
E	0.6078	0.6078	−0.5111
F	0.6078	−0.6078	−0.5111
G	−0.6078	0.6078	−0.5111
H	−0.6078	−0.6078	−0.5111
I	0.0000	0.0000	−1.0000

Separation plane algorithms :

➔ AGE / CDI / BHF

➔ I / EFHG / CBDA

➔ CBF / EHI / ADG

- **SS:9 → Square-face capped square prism**
IUCr symbol : None
IUPAC symbol : CUS-9
Points :

A	−0.5774	−0.5774	−0.5774
B	0.5774	−0.5774	−0.5774
C	−0.5774	0.5774	−0.5774
D	−0.5774	−0.5774	0.5774
E	−0.5774	0.5774	0.5774
F	0.5774	−0.5774	0.5774
G	0.5774	0.5774	−0.5774
H	0.5774	0.5774	0.5774
I	0.0000	0.0000	1.0000

Separation plane algorithms :

➔ BF / ADIHG / CE

➔ I / DEHF / ACGB

➔ BG / ACHF / DEI

- **TO_1:9 → Tricapped octahedron (all 3 cap faces share one atom)**
IUCr symbol : None
IUPAC symbol : TOCT-9
Points :

A	0.0000	0.0000	1.0000
B	0.0000	0.0000	−1.0000
C	1.0000	0.0000	0.0000
D	−1.0000	0.0000	0.0000
E	0.0000	1.0000	0.0000
F	0.0000	−1.0000	0.0000
G	0.5774	0.5774	0.5774
H	0.5774	−0.5774	0.5774
I	0.5774	−0.5774	−0.5774

Separation plane algorithms :

➔ IBF / CDH / GEA

➔ BE / CGDI / AFH

➔ DF / AHIB / GCE

- **TO_2:9 → Tricapped octahedron (cap faces are aligned)**
  IUCr symbol : None
  IUPAC symbol : TOCT-9
  Points :

A	0.0000	0.0000	1.0000
B	0.0000	0.0000	−1.0000
C	1.0000	0.0000	0.0000
D	−1.0000	0.0000	0.0000
E	0.0000	1.0000	0.0000
F	0.0000	−1.0000	0.0000
G	0.5774	0.5774	0.5774
H	0.5774	−0.5774	0.5774
I	−0.5774	−0.5774	−0.5774

Separation plane algorithms :

➔ CB / EGHFI / AD

➔ EB / CGDI / HAF

- **TO_3:9 → Tricapped octahedron (all 3 cap faces are sharingone edge of a face)**
  IUCr symbol : None
  IUPAC symbol : TOCT-9
  Points :

A	0.0000	0.0000	1.0000
B	0.0000	0.0000	−1.0000
C	1.0000	0.0000	0.0000
D	−1.0000	0.0000	0.0000
E	0.0000	1.0000	0.0000
F	0.0000	−1.0000	0.0000
G	0.5774	0.5774	0.5774
H	−0.5774	0.5774	−0.5774
I	0.5774	−0.5774	−0.5774

Separation plane algorithms :

➔ CGA / FIE / BHD

➔ AF / DGCI / HEB

Coordination 10

- **PP:10 → Pentagonal prism**
  IUCr symbol : None
  IUPAC symbol : PPR-10
  Points :

A	1.0000	0.0000	−0.5878
B	0.3090	0.9511	−0.5878
C	−0.8090	0.5878	−0.5878
D	−0.8090	−0.5878	−0.5878
E	0.3090	−0.9511	−0.5878
F	1.0000	0.0000	0.5878
G	0.3090	0.9511	0.5878
H	−0.8090	0.5878	0.5878
I	−0.8090	−0.5878	0.5878
J	0.3090	−0.9511	0.5878

Separation plane algorithms :

➔ ∅ / ABCDE / FGHIJ

➔ BG / ACHF / EDIJ

- **PA:10 → Pentagonal antiprism**
  IUCr symbol : None
  IUPAC symbol : PAPR-10
  Points :

A	1.0000	0.0000	−0.4253
B	0.3090	0.9511	−0.4253
C	−0.8090	0.5878	−0.4253
D	−0.8090	−0.5878	−0.4253
E	0.3090	−0.9511	−0.4253
F	0.8090	0.5878	0.4253
G	−0.3090	0.9511	0.4253
H	−1.0000	0.0000	0.4253
I	−0.3090	−0.9511	0.4253
J	0.8090	−0.5878	0.4253

Separation plane algorithms :

➔ ∅ / ABCDE / FGHIJ

➔ DIH / CEJG / BAF

- **SBSA:10 → Square-face bicapped square antiprism**
  IUCr symbol : None
  IUPAC symbol : SAPRS-10
  Points :

A	0.0000	0.8595	0.5111
B	0.0000	−0.8595	0.5111
C	0.8595	0.0000	0.5111
D	−0.8595	0.0000	0.5111
E	0.6078	0.6078	−0.5111
F	0.6078	−0.6078	−0.5111
G	−0.6078	0.6078	−0.5111
H	−0.6078	−0.6078	−0.5111
I	0.0000	0.0000	−1.0000
J	0.0000	0.0000	1.0000

Separation plane algorithms :

➔ CFE / AJBI / DHG

➔ JBC / ADF / GHIE

- **MI:10 → Metabidiminished icosahedron**
  IUCr symbol : None
  IUPAC symbol : None
  Points :

A	0.0000	0.5257	0.8507
B	0.0000	0.5257	−0.8507
C	0.0000	−0.5257	−0.8507
D	0.5257	0.8507	0.0000
E	0.5257	−0.8507	0.0000
F	−0.5257	−0.8507	0.0000
G	0.8507	0.0000	0.5257
H	−0.8507	0.0000	0.5257
I	−0.8507	0.0000	−0.5257
J	0.8507	0.0000	−0.5257

  Separation plane algorithms :

  ➔ FGE / AJCH / IDB

  ➔ AH / GDIF / JBCE

- **BS_1:10 → Bicapped square prism (opposite faces)**
  IUCr symbol : None
  IUPAC symbol : CUS-10
  Points :

A	−0.5774	−0.5774	−0.5774
B	0.5774	−0.5774	−0.5774
C	−0.5774	0.5774	−0.5774
D	−0.5774	−0.5774	0.5774
E	−0.5774	0.5774	0.5774
F	0.5774	−0.5774	0.5774
G	0.5774	0.5774	−0.5774
H	0.5774	0.5774	0.5774
I	0.0000	0.0000	−1.0000
J	0.0000	0.0000	1.0000

  Separation plane algorithms :

  ➔ FB / ADJHGI / EC

  ➔ ∅ / ABFD / CIGHJE

- **BS_2:10 → Bicapped square prism (adjacent faces)**
  IUCr symbol : None
  IUPAC symbol : CUS-10
  Points :

A	−0.5774	−0.5774	−0.5774
B	0.5774	−0.5774	−0.5774
C	−0.5774	0.5774	−0.5774
D	−0.5774	−0.5774	0.5774
E	−0.5774	0.5774	0.5774
F	0.5774	−0.5774	0.5774
G	0.5774	0.5774	−0.5774
H	0.5774	0.5774	0.5774
I	1.0000	0.0000	0.0000
J	0.0000	1.0000	0.0000

  Separation plane algorithms :

  ➔ BFI / ADHG / CEJ

  ➔ AD / EFBC / JHIG

  ➔ I / BFHG / ADEJC

- **TBSA:10 → Trigonal-face bicapped square antiprism**
  IUCr symbol : None
  IUPAC symbol : SAPRT-10
  Points :

A	0.0000	0.8595	0.5111
B	0.0000	−0.8595	0.5111
C	0.8595	0.0000	0.5111
D	−0.8595	0.0000	0.5111
E	0.6078	0.6078	−0.5111
F	0.6078	−0.6078	−0.5111
G	−0.6078	0.6078	−0.5111
H	−0.6078	−0.6078	−0.5111
I	0.0000	0.9710	−0.2391
J	0.0000	−0.9710	−0.2391

  Separation plane algorithms :

  ➔ DHG / ABJI / CFE

  ➔ ∅ / ACBD / IEFJHG

Coordination 11

- **PCPA:11 → Pentagonal-face capped pentagonal antiprism**
  IUCr symbol : None
  IUPAC symbol : PPRP-11
  Points :

A	1.0000	0.0000	−0.5878
B	0.3090	0.9511	−0.5878
C	−0.8090	0.5878	−0.5878
D	−0.8090	−0.5878	−0.5878
E	0.3090	−0.9511	−0.5878
F	1.0000	0.0000	0.5878
G	0.3090	0.9511	0.5878
H	−0.8090	0.5878	0.5878
I	−0.8090	−0.5878	0.5878
J	0.3090	−0.9511	0.5878
K	0.0000	0.0000	1.0000

  Separation plane algorithms :

  ➔ K / FGHIJ / ABCDE

  ➔ BGHC / AFK / EJID

- **H:11 → Hendecahedron**
IUCr symbol : None
IUPAC symbol : None
Points :

A	0.0000	0.0000	2.0000
B	2.0000	1.0000	1.0000
C	0.0000	−1.0000	1.0000
D	−2.0000	1.0000	1.0000
E	0.0000	2.0000	0.0000
F	1.0000	−1.0000	0.0000
G	−1.0000	−1.0000	0.0000
H	2.0000	1.0000	−1.0000
I	0.0000	−1.0000	−1.0000
J	−2.0000	1.0000	−1.0000
K	0.0000	0.0000	−2.0000

Separation plane algorithm :
➔ DGJ / EACIK / BFH

- **DI:11 → Diminished icosahedron**
IUCr symbol : None
IUPAC symbol : None
Points :

A	0.0000	−1.0000	−1.6180
B	0.0000	1.0000	−1.6180
C	0.0000	−1.0000	1.6180
D	0.0000	1.0000	1.6180
E	−1.0000	−1.6180	0.0000
F	1.0000	−1.6180	0.0000
G	−1.0000	1.6180	0.0000
H	1.0000	1.6180	0.0000
I	−1.6180	0.0000	−1.0000
J	−1.6180	0.0000	1.0000
K	1.6180	0.0000	−1.0000

Separation plane algorithms :
➔ I / GJEAB / DCFKH
➔ FHK / ACDB / EJGI

Coordination 12

- **I:12 → Icosahedron**
IUCr symbol : [12i]
IUPAC symbol : IC-12
Points :

A	0.0000	−1.0000	−1.6180
B	0.0000	1.0000	−1.6180
C	0.0000	−1.0000	1.6180
D	0.0000	1.0000	1.6180
E	−1.0000	−1.6180	0.0000
F	1.0000	−1.6180	0.0000
G	−1.0000	1.6180	0.0000
H	1.0000	1.6180	0.0000
I	−1.6180	0.0000	−1.0000
J	−1.6180	0.0000	1.0000
K	1.6180	0.0000	−1.0000
L	1.6180	0.0000	1.0000

Separation plane algorithm :
➔ EIGJ / ABDC / FKHL

- **PBP:12 → Pentagonal-face bicapped pentagonal prism**
IUCr symbol : None
IUPAC symbol : PPRP-12
Points :

A	1.0000	0.0000	−0.5878
B	0.3090	0.9511	−0.5878
C	−0.8090	0.5878	−0.5878
D	−0.8090	−0.5878	−0.5878
E	0.3090	−0.9511	−0.5878
F	1.0000	0.0000	0.5878
G	0.3090	0.9511	0.5878
H	−0.8090	0.5878	0.5878
I	−0.8090	−0.5878	0.5878
J	0.3090	−0.9511	0.5878
K	0.0000	0.0000	−1.0000
L	0.0000	0.0000	1.0000

Separation plane algorithm :
➔ EJID / AFLK / BGHC

- **TT:12 → Truncated tetrahedron**
IUCr symbol : [12tt]
IUPAC symbol : None
Points :

A	−0.5774	0.5774	−1.7321
B	0.5774	−0.5774	−1.7321
C	−0.5774	−1.7321	0.5774
D	0.5774	−1.7321	−0.5774
E	1.7321	0.5774	0.5774
F	1.7321	−0.5774	−0.5774
G	−1.7321	−0.5774	0.5774
H	−1.7321	0.5774	−0.5774
I	0.5774	1.7321	0.5774
J	−0.5774	1.7321	−0.5774
K	0.5774	0.5774	1.7321
L	−0.5774	−0.5774	1.7321

Separation plane algorithms :

➔ CD / GLFB / KIEAJH

➔ BDF / ACE / HGLKIJ

- **C:12 → Cuboctahedron**
  IUCr symbol : [12co]
  IUPAC symbol : None
  Points :

A	0.0000	−1.0000	−1.0000
B	0.0000	1.0000	−1.0000
C	0.0000	−1.0000	1.0000
D	0.0000	1.0000	1.0000
E	−1.0000	−1.0000	0.0000
F	1.0000	−1.0000	0.0000
G	−1.0000	1.0000	0.0000
H	1.0000	1.0000	0.0000
I	−1.0000	0.0000	−1.0000
J	−1.0000	0.0000	1.0000
K	1.0000	0.0000	−1.0000
L	1.0000	0.0000	1.0000

Separation plane algorithms :

➔ BKH / AFLDGI / ECJ

➔ IGJE / ABDC / KHLF

- **AC:12 → Anticuboctahedron**
  IUCr symbol : [12aco]
  IUPAC symbol : None
  Points :

A	1.0000	0.0000	0.0000
B	0.5000	0.8660	0.0000
C	−0.5000	0.8660	0.0000
D	−1.0000	0.0000	0.0000
E	−0.5000	−0.8660	0.0000
F	0.5000	−0.8660	0.0000
G	0.5000	0.2887	−0.8165
H	−0.5000	0.2887	−0.8165
I	0.0000	−0.5774	−0.8165
J	0.5000	0.2887	0.8165
K	−0.5000	0.2887	0.8165
L	0.0000	−0.5774	0.8165

Separation plane algorithm :

➔ GHI / ABCDEF / JKL

- **SC:12 → Square cupola**
  IUCr symbol : None
  IUPAC symbol : None
  Points :

A	0.9239	0.3827	0.0000
B	0.3827	0.9239	0.0000
C	−0.3827	0.9239	0.0000
D	−0.9239	0.3827	0.0000
E	−0.9239	−0.3827	0.0000
F	−0.3827	−0.9239	0.0000
G	0.3827	−0.9239	0.0000
H	0.9239	−0.3827	0.0000
I	0.5054	0.0000	0.8409
J	0.0000	0.5054	0.8409
K	−0.5054	0.0000	0.8409
L	0.0000	−0.5054	0.8409

Separation plane algorithm :

➔ ∅ / ABCDEFGH / IJKL

- **HP:12 → Hexagonal prism**
  IUCr symbol : [12p]
  IUPAC symbol : HPR-12
  Points :

A	1.0000	0.0000	−0.5000
B	0.5000	0.8660	−0.5000
C	−0.5000	0.8660	−0.5000
D	−1.0000	0.0000	−0.5000
E	−0.5000	−0.8660	−0.5000
F	0.5000	−0.8660	−0.5000
G	1.0000	0.0000	0.5000
H	0.5000	0.8660	0.5000
I	−0.5000	0.8660	0.5000
J	−1.0000	0.0000	0.5000
K	−0.5000	−0.8660	0.5000
L	0.5000	−0.8660	0.5000

Separation plane algorithms :

➔ ∅ / ABCDEF / GHIJKL

➔ FEKL / ADJG / BCIH

- **HA:12 → Hexagonal antiprism**
  IUCr symbol : None
  IUPAC symbol : HAPR-12
  Points :

A	1.0000	0.0000	−0.4278
B	0.5000	0.8660	−0.4278
C	−0.5000	0.8660	−0.4278
D	−1.0000	0.0000	−0.4278
E	−0.5000	−0.8660	−0.4278
F	0.5000	−0.8660	−0.4278
G	0.8660	0.5000	0.4278
H	0.0000	1.0000	0.4278
I	−0.8660	0.5000	0.4278
J	−0.8660	−0.5000	0.4278
K	0.0000	−1.0000	0.4278
L	0.8660	−0.5000	0.4278

  Separation plane algorithm :
  ➔ ∅ / ABCDEF / GHIJKL

Coordination 13

- **SH:13 → Square-face capped hexagonal prism**
  IUCr symbol : None
  IUPAC symbol : None
  Points :

A	1.0000	0.0000	−0.5000
B	0.5000	0.8660	−0.5000
C	−0.5000	0.8660	−0.5000
D	−1.0000	0.0000	−0.5000
E	−0.5000	−0.8660	−0.5000
F	0.5000	−0.8660	−0.5000
G	1.0000	0.0000	0.5000
H	0.5000	0.8660	0.5000
I	−0.5000	0.8660	0.5000
J	−1.0000	0.0000	0.5000
K	−0.5000	−0.8660	0.5000
L	0.5000	−0.8660	0.5000
M	0.9682	−0.5590	0.0000

  Separation plane algorithm :
  ➔ ∅ / ABCDEF / GHIJKLM
