# Supplementary material for: ChemEnv: a fast and robust coordination environment identification tool
Source: Acta Crystallogr B Struct Sci Cryst Eng Mater. 2020 Jul 21;76(Pt 4):683–95. doi: 10.1107/S2052520620007994 (PMC7412753; doi:10.1107/S2052520620007994)
Supplement: Supplementary file 2 [file b-76-00683-sup1.pdf]

1 Introduction to the tutorial

This tutorial will show you how to use the *ChemEnv* package to analyze chemical coordination environments and to identify model coordination environments that are closest to the coordination environment at hand. The latter is decided by the calculation of continous symmetry measures (CSM). A jupyter-notebook version of this tutorial can also be found in the supplementary information.
The analysis of the coordination environments proceeds as follows:

1. Search for neighbors by a modified Voronoi analysis on a grid of distance and angular parameters

2. Calculation of corresponding continous symmetry measures (CSMs) with model environments for all distance and angular parameters

3. Refinement of the results with different strategies (e.g., by using certain distance and angular parameters)

More details can be found in the main article.

2 The very first steps

1. Download and install *pymatgen* (see http://pymatgen.org/ for more information)

2. Sign up for the *Materials Project* on https://materialsproject.org/.

3. Setup the connection to the *Materials Project* in your terminal with:

```
$ pmg config --add PMG_MAPI_KEY <USER_API_KEY>
```

3 Import the relevant modules

Let us start by importing the relevant modules.

```python
from pymatgen.analysis.chemenv.coordination_environments.coordination_geometry_finder \
 import LocalGeometryFinder
import logging
from pymatgen.ext.matproj import MPRester
from pymatgen.analysis.chemenv.coordination_environments.chemenv_strategies import \
 SimplestChemenvStrategy, MultiWeightsChemenvStrategy
from pymatgen.analysis.chemenv.coordination_environments.structure_environments \
 import LightStructureEnvironments
```

4 Load a crystal structure

Next, we will load the crystal structure of our interest. We will start with a very simple example here: α-quartz which is depicted in Figure 1

```
# Get a structure from the Materials Project with the help of the Materials Project ID
a = MPRester()
struct = a.get_structure_by_material_id('mp-7000')
```

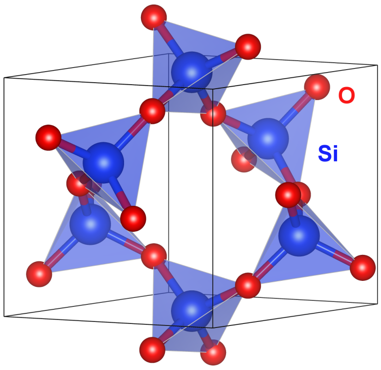

Figure 1: α-Quartz with possible coordination polyhedra (marked in blue). The structure was drawn with VESTA.[1]

Alternatively, one can load the structure from a cif-file:

```
from pymatgen.io.cif import CifParser
parser = CifParser("mystructure.cif")
struct = parser.get_structures()[0]
```

5 Setup of the local geometry finder

First, one has to choose a referene point for the Voronoi analysis. The two relevant parameters are `centering_type` and `include_central_site_in_centroid`. `centering_type` can be either `'standard'`, `'centroid'`, or `'central_site'`. The first results in a default setting, the second one sets the reference point at the centroid of the structure (calculated with or without the site for which the Voronoi analysis is currently performed depending on the Boolean value of `include_central_site_in_centroid`). Have a look at the code for more information. Moreover, a logging is introduced. This is especially important if one runs very large calculations (large distances and small angle parameters) and one wants to access the status of the calculation.

```
# Setup the local geometry finder
lgf=LocalGeometryFinder()
lgf.setup_parameters(centering_type='centroid', include_central_site_in_centroid=True)
#you can also save the logging to a file, just remove the comment
logging.basicConfig(#filename='chemenv_structure_environments.log',
                    format='%(levelname)s:%(module)s:%(funcName)s:%(message)s',
                    level=logging.DEBUG)
lgf.setup_structure(structure=struct)
```

6 Get the StructureEnvironments (=Voronoi analysis plus CSM calculation on a grid of distance and angular parameters)

In the first step, sets of neighbors for different distance and solid angle parameters via Voronoi analysis and corresponding continous symmetry measures (CSMs) are computed. Have a close look at the documentation of the code for more information on parameters that you can define. For example, we will use the `maximum_distance_factor` to save some computing time. Multiplied with the distance to the closest neighbour it results in the maximum distance that is considered in the Voronoi approach. Similarily, one can define another parameter - the `minimum_angle_factor`. This factor is multiplied with the maximum solid angle to the neighbours to arrive at the minimum solid angle that is considered in the Voronoi analysis. Don't choose to drastic values, please. This might lead to unexpected results. One can also refine these parameters later. For more information on these parameters, please consult the main text of the article again. Moreover, one can also exclude atoms from the computation of the coordination environments (e.g., `se = lgf.compute_structure_environments(excluded_atoms =['O'])`). Additional parameters such as `only_atoms`, `only_indices`, and `only_cations` in combination with `valences` might be helpful as well.

```
# Get the StructureEnvironments
se = lgf.compute_structure_environments(maximum_distance_factor=1.41, \
 only_cations=False)
```

We will get an output that shows the proceeding of the Voronoi analysis and the calculation of the `structure_environments`.

7 Different strategies to analyze the StructureEnvironments

Now, the strategy to interpret the data from before is chosen to arrive at information about the coordination environments. One can choose between two different types of strategies: we start with the `SimplestChemenvStrategy`. This strategy type uses fixed angle and distance parameters for the definition of neighbors in the Voronoi approach. The resulting coordination environment is uniquely defined and is then given as the one with the lowest continuous symmetry measure. One of the disadvantages is that it fails for intermediate coordination environments and depends very much on the cutoff parameters chosen.
Important parameters for this strategy are: `distance_cutoff` and `angle_cutoff`. The strategy is correct in about 85% of the cases if one uses `distance_cutoff=1.4` and `angle_cutoff=0.3`. The neighbouring atoms that are considered in this approach have a maximum distance of `distance_cutoff` $\times$ the distance to the closest neighbour and a minimum solid angle of `angle_cutoff` $\times$ the biggest solid angle.

```
strategy=SimplestChemenvStrategy(distance_cutoff=1.4, angle_cutoff=0.3)
lse=LightStructureEnvironments.from_structure_environments(strategy=strategy, \
 structure_environments=se)
```

Next, one can print the information on the coordination environments for each site in the

structure. Here, the information for a site occupied by one oxygen is printed. Please be aware that the counting of the sites starts at 0. In case of doubt, please print the respective site of the `structure` object with `print(structure[isite])` where `isite` is an Integer.

```
#print coordination environments for a special site
isite=5
print(lse.coordination_environments[isite])
```

It will return a dictionary including all relevant information:

```
[{'ce_symbol': 'A:2', 'ce_fraction': 1.0, 'csm': 2.2602837757388414,
 ↪'permutation': [0, 1]}]
```

The item corresponding to the key `'ce_symbol'` symbolizes the coordination environment. `'A:2 '` represents the angular coordination environment. The full list of coordination environments can be seen in the supporting information of this article. With this strategy, the value of `'ce_fraction'` is always equal to 1.0. The `'csm'` represents the continous symmetry measure. This value lies between 0.0 and 100.0. At 0.0 the chemical environment in the structure is identical to the model environment and can be interpreted as a distance to a shape. In this example, a `'csm'` of 2.26 shows that the environment still shows some similarity to the model environment. Coordination environments with an `'csm'` greater than 2.5 are already considered as rather distorted. For more information on the csm, have a look at the main text.
A more evolved strategy type, especially for intermediate coordination enviroments, is the `MultiWeightsChemenvStrategy`. In the following, the default parameters (weigths) are used. Of course, experts can also modify these weights.

```
#Get the strategy from D. Waroquiers et al., Chem Mater., 2017, 29, 8346.
from pymatgen.analysis.chemenv.coordination_environments.chemenv_strategies import \
 MultiWeightsChemenvStrategy
strategy = MultiWeightsChemenvStrategy.stats_article_weights_parameters()
lse = LightStructureEnvironments.from_structure_environments(strategy=strategy, \
 structure_environments=se)
```

We will start with the same oxygen site as before:

```
#print coordination environments for a special site
isite = 5
print( lse.coordination_environments[isite] )
```

This will result in the following output:

```
[{'ce_symbol': 'A:2', 'ce_fraction': 0.6735414322036577, 'csm':
 ↪2.2602837757388414, 'permutation': [0, 1]}, {'ce_symbol': 'L:2',
 ↪'ce_fraction': 0.3264585677963424, 'csm': 2.8902708777516026,
 ↪'permutation': [0, 1]}]
```

This time, two coordination environments for one site exist as indicated by two appearances of `ce_symbol` and there is also a `ce_fraction` for each of the environments. The latter indicates the coordination environment is an intermediate between `'A:2'` (angular) and `'L:2'` (linear) with 67% angular environment and 33% linear environment. Also, there is a `csm` value for

both environments. As already indicated by the `ce_fraction`, the csm for `'A:2'` is lower (=in better agreement with the model environment) than the csm for `'L:2'`.
Now, an example follows where only one coordination environment exists. A Si occupies the corresponding site.

```
#another site where you have only one coordination environment (tetrahedron, T:4)
isite = 1
print( lse.coordination_environments[isite] )
```

We will receive the following output:

```
[{'ce_symbol': 'T:4', 'ce_fraction': 1.0, 'csm': 0.009887786111312944,
↪'permutation': [0, 1, 2, 3]}]
```

The resulting coodination environment is `'T:4'` (tetrahedron).

8 References

[1] K. Momma, F. Izumi, VESTA3 for Three-Dimensional Visualization of Crystal, Volumetric and Morphology Data, J. Appl. Crystallogr. 44 (6) (2011) 1272–1276. doi:DOI: 10.1107/S0021889811038970.
